# Differential stability of Gcn4p controls its cell-specific activity in differentiated yeast colonies

**DOI:** 10.1128/mbio.00689-24

**Published:** 2024-04-16

**Authors:** Libuše Váchová, Vítězslav Plocek, Jana Maršíková, Stanislava Rešetárová, Ladislava Hatáková, Zdena Palková

**Affiliations:** 1 Institute of Microbiology of the Czech Academy of Sciences, BIOCEV, Prague, Czech Republic; 2 Faculty of Science, Charles University, BIOCEV, Prague, Czech Republic; Harvard Medical School, Boston, Massachusetts, USA

**Keywords:** differentiated colonies, spatially structured populations, cell-specific regulation, proteasomal degradation, transcription factor, yeast, *Saccharomyces cerevisiae*

## Abstract

Gcn4p belongs to conserved AP-1 transcription factors involved in many cellular processes, including cell proliferation, stress response, and nutrient availability in yeast and mammals. AP-1 activities are regulated at different levels, such as translational activation or protein degradation, which increases the variability of regulation under different conditions. Gcn4p activity in unstructured yeast liquid cultures increases upon amino acid deficiency and is rapidly eliminated upon amino acid excess. Gcn2p kinase is the major described regulator of Gcn4p that enables *GCN4* mRNA translation *via* the uORFs mechanism. Here, we show that Gcn4p is specifically active in U cells in the upper regions and inactive in L cells in the lower regions of differentiated colonies. Using *in situ* microscopy in combination with analysis of mutants and strains with GFP at different positions in the translational regulatory region of Gcn4p, we show that cell-specific Gcn4p activity is independent of Gcn2p or other translational or transcriptional regulation. Genetically, biochemically, and microscopically, we identified cell-specific proteasomal degradation as a key mechanism that diversifies Gcn4p function between U and L cells. The identified regulation leading to active Gcn4p in U cells with amino acids and efficient degradation in starved L cells differs from known regulations of Gcn4p in yeast but shows similarities to the activity of AP-1 ATF4 in mammals during insulin signaling. These findings may open new avenues for understanding the parallel activities of Gcn4p/ATF4 and reveal a novel biological role for cell type-specific regulation of proteasome-dependent degradation.

**IMPORTANCE:**

In nature, microbes usually live in spatially structured communities and differentiate into precisely localized, functionally specialized cells. The coordinated interplay of cells and their response to environmental changes, such as starvation, followed by metabolic adaptation, is critical for the survival of the entire community. Transcription factor Gcn4p is responsible for yeast adaptation under amino acid starvation in liquid cultures, and its activity is regulated mainly at the level of translation involving Gcn2p kinase. Whether Gcn4p functions in structured communities was unknown. We show that translational regulation of Gcn4p plays no role in the development of colony subpopulations; the main regulation occurs at the level of stabilization of the Gcn4p molecule in the cells of one subpopulation and its proteasomal degradation in the other. This regulation ensures specific spatiotemporal activity of Gcn4p in the colony. Our work highlights differences in regulatory networks in unorganized populations and organized structures of yeast, which in many respects resemble multicellular organisms.

## INTRODUCTION

**Fig 1:**
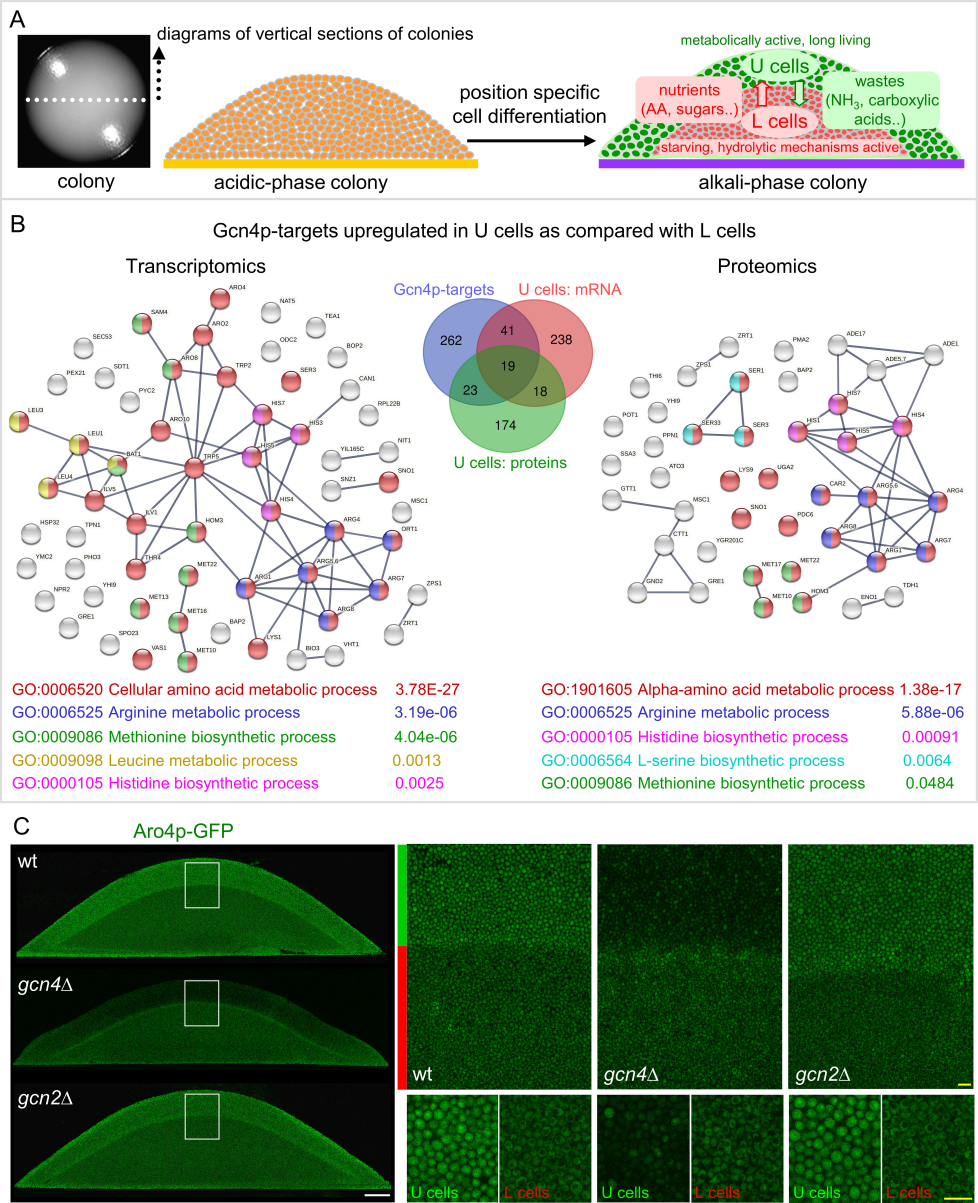
Gcn4p regulates gene expression in U cells independently of Gcn2p. (**A**) Diagrams of U and L cell localization in differentiated colonies, their properties, and interaction. AA, amino acids. (**B**) Gcn4p-regulated targets identified in mRNAs and proteins upregulated in U versus L cells. Venn diagram (in the center) showing calculated intersections of Gcn4p targets, mRNAs upregulated in U versus L cells, and proteins elevated in U versus L cells in the indicated comparisons (list of genes in Table S1A). Functional and direct associations between identified Gcn4p-regulated targets identified with STRING with the highest confidence (0.900) based on transcriptome data (left) and proteome data (right). Functional enrichment revealed highly significant gene ontology (GO) clusters related to amino acid metabolism (Table S1B and C). (**C**) Aro4p-GFP level in U cells is dependent on Gcn4p and independent of Gcn2p. Left, 2PE-CM images of vertical cross-sections of fully differentiated 4-day-old microcolonies of wt-Aro4p-GFP, *gcn4*-Aro4p-GFP, and *gcn2*-Aro4p-GFP strains. Right, magnified areas of colonies (white rectangles in left panels); green and red bars show the position of U and L cell layers (upper panels). Images of U and L cells at higher magnification (lower panels). White bar, 100 μm; yellow bar, 10 μm. Representative results from five independent biological experiments are shown.

Yeast forms colonies in which cellular metabolism is reprogrammed during development and aging, helping colonies cope with the gradual loss of nutrients. *Saccharomyces cerevisiae* colonies on the respiratory medium undergo phases of change in extracellular pH, with the alkaline phase associated with the production of volatile ammonia, which plays a role in metabolic reprogramming as a quorum-sensing signal (1–3). Ammonia signaling is associated with marked differentiation of aging colonies, in which two major, metabolically distinct cell types develop (Fig. 1A): Long-lived U cells are formed in the upper parts of the colony, whereas stressed L cells with reduced viability develop in the middle and lower parts. Both cell types have specific properties and a number of differences (metabolic and regulatory) from yeast cells in chronologically aging liquid cultures (4–8). U cells combine stress resistance, accumulation of storage compounds, and active autophagy with metabolic activity, including active TORC1, glycolysis, and amino acid metabolism, and they contain relatively high levels of intracellular amino acids, such as glutamine and glutamate (6). A number of metabolic and regulatory features of U cells, including aerobic glycolysis, are reminiscent of mammalian tumor cells (6, 9). U cells also reduce mitochondrial respiration and have active mitochondrial retrograde (RTG) signaling (10). By contrast, L cells are metabolically less active, exhibit starvation characteristics, do not accumulate storage compounds, have an inactive TORC1, and instead enhance hydrolytic mechanisms and release cell wall components, which may contribute to their stress sensitivity (6, 11). Metabolites released by L cells are utilized by U cells and are important for their survival.

Transcriptomics of U cells has revealed the expression of amino acid metabolism genes and suggested a function of the transcription factor Gcn4p, although U cells have active TORC1 (6, 8), a negative Gcn4p regulator (12). Yeast Gcn4p (“General Control Nondepressible”) is a pleiotropic transcriptional activator that belongs to the AP-1 (Activating protein-1) basic leucine-zipper family. It functions primarily as an activator of genes involved in amino acid and purine biosynthesis under amino acid limitation (13), but may also link amino acid and glucose metabolism (14, 15). Gcn4p also directly or indirectly regulates the expression of numerous other genes involved in autophagy, stress response, and glycogen metabolism (16) and its binding sites have been identified in promoters of hundreds of different genes (17, 18). Gcn4p recognizes a similar binding site and shares amino acid sequence homology with the mammalian JunC and JunD proteins of the AP-1 family (e.g., DNA-binding domains of Gcn4p and Jun are 45% identical) (18, 19), which consists of four subfamilies (Jun, Fos, ATF/cAMP-responsive element-binding, and Maf families (20, 21)), involved in processes such as proliferation, apoptosis, development, and the response to stress (22, 23). Similar to *ATF4* in mammals (24, 25), *GCN4* mRNA is translated in response to stress and induces the expression of genes involved in stress adaptation. This adaptation, termed the integrated stress response (ISR), is triggered in yeast and mammals by the Gcn2p kinase, which is activated, for example, upon amino acid limitation, and phosphorylates and inhibits the translation initiation factor eIF2α. This reduces the amount of GTP-bound eIF2α and mRNA translation globally, except for a few mRNAs, including *GCN4*/*ATF4*, with small inhibitory ORFs in the 5′-UTR of their mRNA (uORFs) involved in Gcn2p-mediated regulation of translation (26). In the presence of nutrients, after translation of uORF1, the 40S ribosome restarts at uORF2, uORF3, or uORF4, and after termination, the 80S ribosome dissociates and *GCN4* translation is inhibited. After activation of Gcn2p and inhibition of eIF2α, the 40S ribosomes pass uORF4, leading to translation of the major ORF - the *GCN4* gene (27). The function of Gcn2p in yeast is inhibited by TORC1, resulting in the inhibition of *GCN4* translation (28).

In addition to translation, the stability of AP-1 proteins can also be controlled. In the presence of amino acids, Gcn4p is phosphorylated by the Pho85p-cyclin-dependent kinase/Pcl5p-cyclin complex and degraded *via* the SCF (Cdc4p)-ubiquitin ligase complex and proteasomes, but its half-life increases sharply under amino acid starvation (29–33). When methionine is present, the degradation of Gcn4p is also reduced by its dephosphorylation *via* the Ppm1p-methylated phosphatase PP2A (34). In mammals, phosphorylation of the Gcn4p ortholog c-Jun in neuronal cells results in c-Jun being polyubiquitinated and degraded by FBW7 (35), and nuclear ATF4 can also be phosphorylated, ubiquitinated, and sent for degradation (36).

Here we show diversification of Gcn4p activity between U cells (Gcn4p active) and L cells (Gcn4p inactive) that is mediated strictly by differential proteasomal degradation of nuclear Gcn4p, without the above-described involvement of Gcn2p, TORC1, methionine, Ppm1p or amino acids.

## RESULTS AND DISCUSSION

### Gcn4p regulates gene expression in U cells independently of Gcn2p

First, we compared previously identified genes potentially regulated by Gcn4p (https://www.yeastgenome.org/locus/S000000735/regulation) with groups of mRNAs and proteins identified by transcriptomics and proteomics that are upregulated in U cells compared with L cells (6, 37–39). This comparison identified 60 potential Gcn4p targets within mRNAs and 42 targets within proteins upregulated in U cells, with 19 targets identified in both comparisons (Fig. 1B; Table S1A). Gene Ontology (GO) enrichment functional analysis of these mRNAs and proteins revealed a highly conclusive GO category related to amino acid metabolism (Fig. 1B, Table S1B and C), including groups of genes/proteins involved in the metabolism of arginine, methionine, and histidine in both comparisons and mRNAs of genes involved in leucine metabolism and enzymes of serine metabolism in individual comparisons.

Among the identified genes, the *ARO4* gene was previously used as a marker for Gcn4p activity (40), and its mRNA level was higher in U cells than in L cells (Fig. 1B). Moreover, the amount of Aro4p-GFP produced in the wt strain with a C-terminal fusion of the *ARO4* gene with GFP in the genome (wt-Aro4p-GFP, Table 1) was sufficient for microscopic detection on vertical cross-sections of colonies. Therefore, we constructed strains derived from wt-Aro4p-GFP with the *GCN4* or *GCN2* gene deleted (*gcn4*-Aro4p-GFP and *gcn2*-Aro4p-GFP, Table 1) and used Aro4p-GFP as a marker to determine the activity of Gcn4p directly *in situ* in colonies. We examined Aro4p-GFP levels in 4-day-old microcolonies that had differentiated into U and L cells by confocal microscopy with two-photon excitation (2PE-CM) of colony cross-sections (Fig. 1C). Aro4p-GFP fluorescence was present in both U and L cells of wt-Aro4p-GFP colonies, but deletion of the *GCN4* gene resulted in a strong decrease in Aro4p-GFP only in U cells. Aro4p expression in L cells was not affected by *GCN4* deletion, and the absence of the Gcn2p kinase did not affect Aro4p-GFP levels in any cell.

**TABLE 1:** *S. cerevisiae* strains

Strain	Genotype	Source
wt (BY4742)	MATα, his3Δ1, leu2Δ0, lys2Δ0, ura3Δ0	Euroscarf
wt-Aro4p-GFP	MATα, his3Δ1, leu2Δ0, lys2Δ0, ura3Δ0, ARO4-yEGFP-KanMX	This study
*gcn4*-Aro4p-GFP	MATα, his3Δ1, leu2Δ0, lys2Δ0, ura3Δ0, gcn4::NatMX, ARO4-yEGFP-KanMX	This study
*gcn2*-Aro4p-GFP	MATα, his3Δ1, leu2Δ0, lys2Δ0, ura3Δ0, gcn2::NatMX, ARO4-yEGFP-KanMX	This study
nAuORF2-GFP	MATα, his3Δ1, leu2Δ0, lys2Δ0, ura3Δ0, nAuORF2-yEGFP-KanMX-GCN4	This study
uORF1-GFP	MATα, his3Δ1, leu2Δ0, lys2Δ0, ura3Δ0, uORF1-yEGFP-KanMX-GCN4	This study
uORF2-GFP	MATα, his3Δ1, leu2Δ0, lys2Δ0, ura3Δ0, uORF2-yEGFP-KanMX-GCN4	This study
uORF3-GFP	MATα, his3Δ1, leu2Δ0, lys2Δ0, ura3Δ0, uORF3-yEGFP-KanMX-GCN4	This study
uORF4-GFP	MATα, his3Δ1, leu2Δ0, lys2Δ0, ura3Δ0, uORF4-yEGFP-KanMX-GCN4	This study
p_GCN4_-GFP	MATα, his3Δ1, leu2Δ0, lys2Δ0, ura3Δ0, gcn4::yEGFP-KanMX	This study
wt-Gcn4p-GFP	MATα, his3Δ1, leu2Δ0, lys2Δ0, ura3Δ0, GCN4-yEGFP-KanMX	This study
wt-Gcn4p-HA	MATα, his3Δ1, leu2Δ0, lys2Δ0, ura3Δ0, GCN4-6HA-KanMX	This study
*gcn2*-Gcn4p-GFP	MATα, his3Δ1, leu2Δ0, lys2Δ0, ura3Δ0, gcn2::NatMX, GCN4-yEGFP-KanMX	This study
wt-p_TEF_-Gcn4p-GFP	MATα, his3Δ1, leu2Δ0, lys2Δ0, ura3Δ0, NatMX-p_TEF_-GCN4-yEGFP-KanMX	This study
wt-p_TEF_-Gcn4p-HA	MATα, his3Δ1, leu2Δ0, lys2Δ0, ura3Δ0, NatMX-p_TEF_-GCN4-6HA-KanMX	This study
*pcl5*-Gcn4p-HA	MATα, his3Δ1, leu2Δ0, lys2Δ0, ura3Δ0, pcl5::HphMX, NatMX-p_TEF_-GCN4-6HA-KanMX	This study
*rpn13*-Gcn4p-HA	MATα, his3Δ1, leu2Δ0, lys2Δ0, ura3Δ0, rpn13::HphMX, NatMX-p_TEF_-GCN4-6HA-KanMX	This study
*rpn14*-Gcn4p-HA	MATα, his3Δ1, leu2Δ0, lys2Δ0, ura3Δ0, rpn14::HphMX, NatMX-p_TEF_-GCN4-6HA-KanMX	This study
*ppm1*-p_TEF_-Gcn4p-GFP	MATα, his3Δ1, leu2Δ0, lys2Δ0, ura3Δ0, ppm1::HphMX, NatMX-p_TEF_-GCN4-GFP-KanMX	This study
*sam1*-p_TEF_-Gcn4p-GFP	MATα, his3Δ1, leu2Δ0, lys2Δ0, ura3Δ0, sam1::HphMX, NatMX-p_TEF_-GCN4-GFP-KanMX	This study
*sam2*-p_TEF_-Gcn4p-GFP	MATα, his3Δ1, leu2Δ0, lys2Δ0, ura3Δ0, sam2::HphMX, NatMX-p_TEF_-GCN4-GFP-KanMX	This study
*gcn2*-Gcn4p-HA	MATα, his3Δ1, leu2Δ0, lys2Δ0, ura3Δ0, gcn2::NatMX, GCN4-6HA-KanMX	This study
*gcn2*-p_TEF_-Gcn4p-GFP	MATα, his3Δ1, leu2Δ0, lys2Δ0, ura3Δ0, gcn2::HphMX, NatMX-p_TEF_-GCN4-yEGFP-KanMX	This study

**Fig 2:**
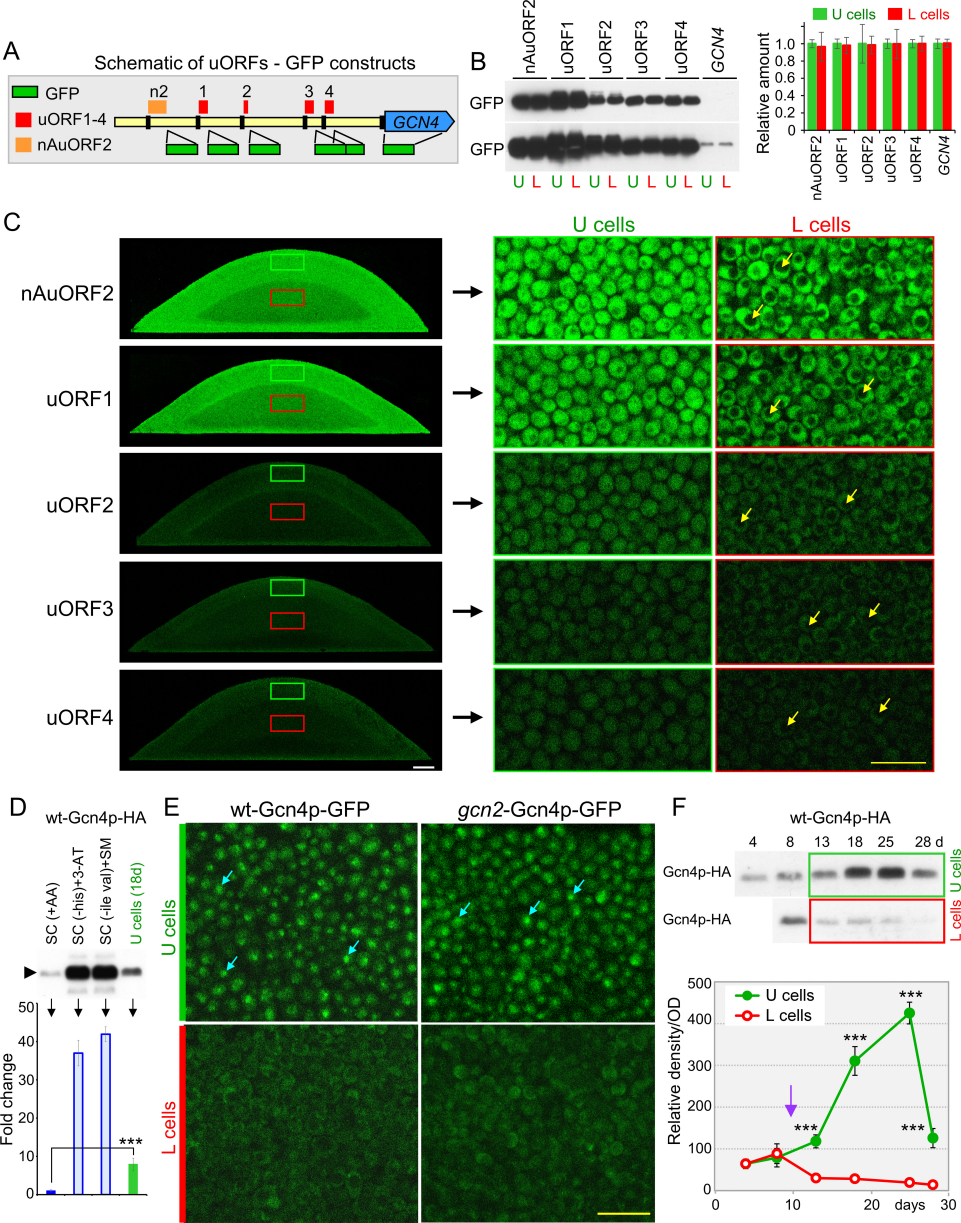
Gcn4p localizes to the nuclei of U cells independently of transcriptional/translational regulation. (**A**) Schematic description of constructed strains with GFP gene fusions with nAuORF2 and individual uORFs1-4 regulating translation of *GCN4* gene, and replacement of *GCN4* coding sequence. (**B**) Western blot (WB) of GFP expressed from nAuORF2, uORFs1-4, and the ATG codon of the *GCN4* coding sequence in U and L cells separated from 15-day-old differentiated giant colonies. The lower panel shows a longer exposure demonstrating that there is no difference in GFP production when GFP replaces the *GCN4* coding sequence. Loading control in Fig. S8A. The graph (quantification of GFP abundance in the WB) shows the relative abundance of GFP in L cells compared to U cells for each strain. Mean values and SD of four western blots from two independent experiments are shown. (**C**) Expression of individual uORFs/*GCN4* ORF in colonies. Left: Cross-sections of 4-day-old microcolonies showing the intensity of cytosolic GFP signal in U and L cells when the GFP gene was fused with nAuORF2 and uORFs1-4, visualized by 2PE-CM. The fluorescence of GFP positioned in place of Gcn4p was below the detection limit of 2PE-CM and is not shown. White bar, 100 μm. Right: Cross-sections of the same microcolonies as in the left panel at higher magnification; examples of vacuoles in L cells are indicated by yellow arrows. Yellow bar, 10 μm. The same 2PE-CM laser intensity was used for all images. (**D**) Western blot of Gcn4p-HA in cells of the wt-Gcn4p-HA strain from liquid cultures in SC medium with all amino acids (SC +AA), in the same medium without histidine and treated with 3-aminotriazole (SC(-his)+3 AT) or in the same medium without isoleucine and valine and treated with sulfometuronmethyl (SC(-ile val)+SM) as well as in U cells from fully differentiated 18-day-old giant colonies. Loading control in Fig. S8B. The graph shows the quantification of the Gcn4p-HA WB signal in different cell types/media as a fold change compared to the signal in cells in SC +AA. Mean values and SD of four WBs from two independent experiments are shown. Statistical significance: ***, *P*-value < 0.001. (**E**) Amount and localization of Gcn4p-GFP in U and L cells of wt and *gcn2*Δ strains. Cellular localization of Gcn4p-GFP in U (green bar) and L (red bar) cells from 4-day-old microcolonies of wt-Gcn4p-GFP and *gcn2*-Gcn4p-GFP strains using 2PE-CM of vertical cross-sections. Shown is the nuclear localization of Gcn4p-GFP in U cells (examples indicated by turquoise arrows). Yellow bar, 10 μm. (**F**) Gcn4p amounts in lysates of upper/U and lower/L cells separated from giant colonies of the wt-Gcn4p-HA strain. Loading control in Fig. S8C. Graph shows quantification of changes in Gcn4p concentration in upper/U (green curve) and lower/L (red curve) cells from giant colonies. Results are given as means and SD of five western blots from two independent experiments. Statistical significance of the difference between U and L cells was determined using an unpaired two-tailed *t*-test and GraphPad Prism 6 software; ***, *P*-value < 0.001. The purple arrow indicates the onset of colony differentiation. Representative results from two (**B, C, D, and F**) and four (**E**) independent experiments are shown.

Translational regulation *via* the Gcn2p kinase is the major regulation of *GCN4* expression described to date (41). We, therefore, constructed a series of strains (Fig. 2A; Table 1) in which GFP has positioned downstream of the short coding sequences and upstream of the stop codons of each of these 4 uORFs and, in addition also downstream of nAuORF2 which starts with a non-AUG codon (nAuORF2). The nAuORF2 is also translated *in vivo* (41). We also constructed a strain in which GFP replaced the coding *GCN4* sequence. To compare the efficiency of translation of these ORFs between U and L cells, we analyzed GFP levels by 2PE-CM on cross-sections of 4-day-old differentiated microcolonies of these strains (Fig. 2C) and by Western blot in lysates of U and L cells separated from 15-day-old differentiated giant colonies (Fig. 2B). As expected, GFP levels were in both colony types highest when the GFP gene was fused to uORF1 (and to nAuORF2), intermediate when GFP was fused to any of uORFs2-4, and very low when GFP replaced the coding *GCN4* sequence. Despite large differences in GFP concentrations depending on the position of GFP relative to the respective ORFs, GFP ratios between U and L cells were nearly identical in all cases, as shown by Western blot (Fig. 2B) and GFP fluorescence intensity in the cytosol of cells of the respective cell subpopulations (Fig. 2C).

These data showed that the differential activity of Gcn4p in U and L cells, as demonstrated by Gcn4p-dependent Aro4p-GFP expression specific to U cells, is not regulated by the mechanism commonly associated with amino acid deficiency—the induction of *GCN4* mRNA translation by Gcn2p/phospho-eIF2α in starving cells (13). This conclusion is based on the finding that Aro4p-GFP expression in U cells is Gcn4p-dependent and Gcn2p-independent (Fig. 1C) and that uORFs regulate translation of the GFP reporter similarly in U and L cells (Fig. 2A through C).

U cells have a specific metabolism and regulation that differs from both starving cells and proliferating cells grown with nutrients (3, 6, 8). Gcn4p levels are strongly induced in cells deprived of nutrients (e.g., amino acids) in liquid cultures depending on Gcn2p kinase (13, 14, 26). Therefore, we compared the Gcn4p amount in U cells with the Gcn4p amount in starving/non-starving cells in liquid cultures. We grow the wt-Gcn4p-HA strain in an SC medium with amino acids and in two starvation media in which Gcn4p-HA translation is increased by Gcn2p regulation (42). The results (Fig. 2D) show that the Gcn4p-HA content in U cells of 18-day-old, fully differentiated giant colonies is intermediate between non-starved and starved cells of liquid cultures, being about 7.9-fold higher than Gcn4p-HA in non-starved cells and about fivefold lower than Gcn4p-HA in starved cells. Like other features, the characteristics of Gcn4p expression point to the unique metabolic properties of U cells, which form in colonies during starvation but draw nutrients for further slow growth from L cells and from their metabolic reprogramming (e.g., autophagy) (3, 6, 11).

### Gcn4p localizes to the nuclei of U cells independently of transcriptional or translational regulation

Next, we examined whether the level and nuclear localization of Gcn4p correspond to Gcn4p activity in colonies. First, we determined the level and cellular localization of Gcn4p in undifferentiated and differentiated cells by *in situ* colony microscopy using strains with a *GCN4* gene fused to GFP derived from wt (wt-Gcn4p-GFP) and *gcn2*Δ (*gcn2*-Gcn4p-GFP) strains (Table 1). Colonies of the wt strain without GFP were used as autofluorescence control. Gcn4p-GFP was barely detectable in the upper and lower parts of the 3-day-old undifferentiated colonies (Fig. S1A). Very weak Gcn4p-GFP fluorescence was detected only in some cells in the upper part of colonies of both wt-Gcn4p-GFP and *gcn2*-Gcn4p-GFP strains. By contrast, much stronger Gcn4p-GFP fluorescence localized in the nuclei of U cells was detected in differentiated 4-day-old microcolonies of both wt-Gcn4p-GFP and *gcn2*-Gcn4p-GFP strains, whereas GFP fluorescence was undetectable in the nuclei of L cells of both strains (Fig. 2E; Fig. S1B). Compared with the background autofluorescence, GFP could be present in the cytosol of some L cells (Fig. S1B). These results supported the Gcn2p-independent Gcn4p activity specific to U cells and suggested that, in contrast to the absence of transcriptional/translational regulation responsible for the difference between U and L cells, Gcn4p levels are increased in differentiated cells compared to cells in younger undifferentiated colonies.

Overall, Gcn4p-GFP levels were low (at the sensitivity limit of 2PE-CM, especially in undifferentiated 3-day-old microcolonies), making it difficult to distinguish cytosolic Gcn4p-GFP fluorescence. Therefore, we also quantified the differences in the concentrations of Gcn4p-HA in cell subpopulations separated from the upper and lower parts of developing giant colonies of wt-Gcn4p-HA strain (Table 1) by western blot, which showed moderate Gcn4p concentrations in 4–8 day-old giant colonies before differentiation (which begins ~10–12 days in giant colonies), a sharp increase in Gcn4p-HA in U cells, and a gradual decrease in L cells (Fig. 2F). The position of Gcn4p-HA (6 HA tag) in SDS-PAGE was consistent with the position of ~60 kDa previously shown for the same form of Gcn4p-HA (43) and the band was not detected in control cells without HA tag (Fig. S2). These data confirmed the correlation between Gcn4p activity detected by Gcn4p-regulated Aro4p expression and Gcn4p nuclear localization, both of which were specifically high in U cells and negligible in L cells. The identical Gcn4p regulation in microcolonies, which differentiate after ~4 days, and giant colonies, which differentiate after ~10–12 days, confirms the specificity of Gcn4p activity in U cells, whose properties are independent of the timing of chronological aging that differs between microcolonies and giant colonies (8).

Our attempt to compare the Gcn4p-HA concentration in the developing giant colonies of the *gcn2*-Gcn4p-HA strain (Table 1) with the wt-Gcn4p-HA strain by western blot was unsuccessful due to the substantial increase of dead cells in the upper regions of the giant colonies of the *gcn2*-Gcn4p-GFP strain (Fig. S3), even before U/L cell differentiation. This made quantification of Gcn4p-HA in *gcn2*-Gcn4p-GFP giant colonies impossible. This finding suggests a role for the Gcn2p kinase in aged giant colonies, in which aging-related factors are more prominent than in microcolonies (8), but such functions are not associated with U cell-specific Gcn4p, as shown by the same pattern of Gcn4p-GFP in wt-Gcn4p-GFP and *gcn2*-Gcn4p-GFP microcolonies (Fig. 2E).

**Fig 3:**
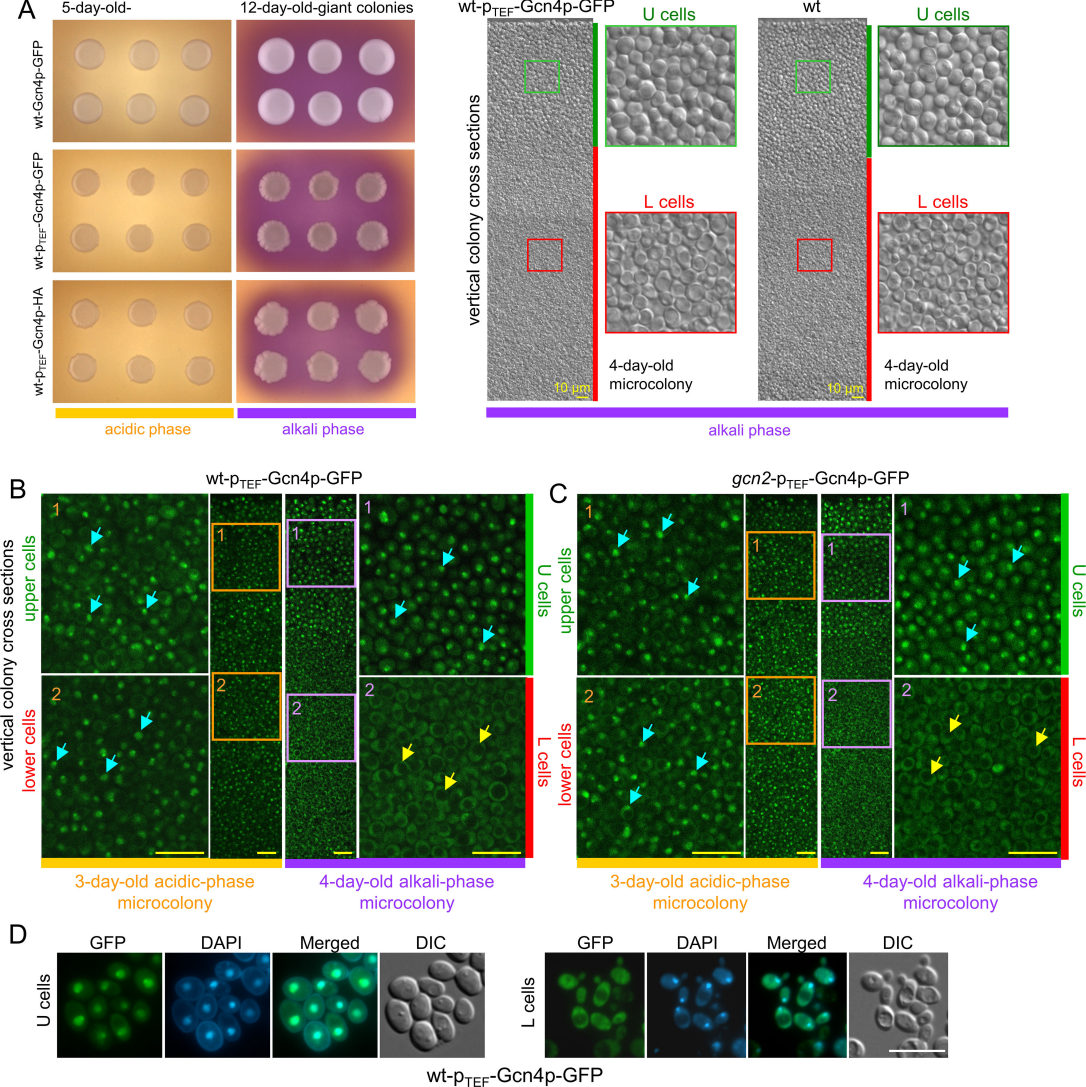
Localization of constitutively overproduced Gcn4p-GFP in colonies. (**A**) Growth and U/L cell differentiation of colonies of p_TEF_-Gcn4p strains. Left: Giant colonies of wt-Gcn4p-GFP, wt-p_TEF_-Gcn4p-GFP, and wt-p_TEF_-Gcn4p-HA strains in acidic and alkaline phases of development grown on GMA-BCP plates. Right: Vertical cross-section of a 4-day-old microcolony in the alkaline phase of the wt-p_TEF_-Gcn4p-GFP strain and wt strain BY4742 (as control). Shown are the correct U/L cell differentiation and the morphology of U and L cells visualized by DIC. Green bar, U cells; red bar, L cells. (**B and C**) Cellular localization of Gcn4p-GFP in colonies of the wt-p_TEF_-Gcn4p-GFP and *gcn2*-p_TEF_-Gcn4p-GFP strain in acidic (left panels, orange bar) and alkaline (right panels, purple bar) phases of their development. The areas of colonies in the acidic phase (orange rectangles; 1, upper and 2, lower cells) and the alkaline phase (purple rectangle; 1, U and 2, L cells) are shown at higher magnification. Yellow bar, 10 μm; turquoise arrows show examples of nuclear localization. Large vacuoles without GFP fluorescence are visible in L cells (examples indicated by yellow arrows). Representative results of three independent biological experiments are shown. A comparison of Gcn4p-GFP fluorescence with background fluorescence is shown in Fig. S4. (**D**) Nuclear and cytosolic localization of Gcn4p-GFP in U and L cells from 4-day-old microcolonies of the wt-p_TEF_-Gcn4p-GFP strain. Cells with Gcn4p-GFP were stained with DAPI to visualize the position of the nucleus. The DIC image shows the morphology of the U and L cells. White bar, 10 μm.

Unlike some other transcription factors, the transport of synthesized Gcn4p into the nucleus is constitutive (32), and the concentration of Gcn4p in the nucleus therefore depends on *GCN4* expression (transcription/translation) and Gcn4p stability. Besides the most common Gcn2p-dependent *GCN4* translation, Gcn2p-independent translational regulation of Gcn4p occurs under certain conditions (26). For example, *GCN4* translation occurred under stress conditions in which tRNAs were modified and accumulated in the nucleus (44, 45). To determine whether a transcription/translation regulation of *GCN4* is somehow involved in colonies, we constructed strains with increased constitutive production of Gcn4p by placing a moderately strong *TEF1* promoter (p_TEF_) in the genome immediately upstream of *GCN4* ORF. p_TEF_ provides nearly equal gene expression in U and L cells (8), and the construct precluded regulation by the native *GCN4* promoter or uORFs. Overproduction of Gcn4p-GFP also allows better visualization of cellular localization of Gcn4p-GFP *in situ* in cross-sections of developing colonies. Attempts to construct a wt-p_TEF_-Gcn4p strain (without GFP or HA tag) were unsuccessful, suggesting that higher concentrations and/or constitutive expression of Gcn4p are toxic to yeast cells. This is consistent with the previously reported toxicity of high concentrations of Gcn4p (33, 46). However, we were able to place p_TEF_ upstream of *GCN4*-GFP (wt-p_TEF_-Gcn4p-GFP strain) and *GCN4*-HA (wt-p_TEF_-Gcn4p-HA strain) (Table 1), suggesting that GFP and HA tags at the C-terminus of Gcn4p slightly reduce the functionality of Gcn4p and therefore partially suppress the toxic effect of *GCN4* overexpression. Colonies of these strains exhibited a partial growth defect but showed proper differentiation into U and L cells (Fig. 3A). In young, undifferentiated wt-p_TEF_-Gcn4p-GFP colonies, constitutively expressed Gcn4p-GFP was localized in the nuclei of all cells (Fig. 3B, left panels), whereas in differentiated colonies, Gcn4p-GFP was present in the nuclei (and cytosol) of U cells and only in the cytosol of L cells (Fig. 3B, right panels), similar to Gcn4p-GFP expressed from the native promoter (Fig. 2E). This cellular pattern of constitutively overexpressed Gcn4p-GFP was identical in the colonies of the *gcn2*-p_TEF_-Gcn4p-GFP strain (Fig. 3C). Nuclear/cytosolic localization of Gcn4p-GFP was confirmed by staining the nuclei of U and L cells with DAPI (Fig. 3D).

Taken together, neither transcriptional nor translational regulation plays a role in the differences in nuclear localization and activity of Gcn4p between U and L cells in colonies. Two sets of data support this: First, the same amount of GFP is produced in U and L cells in which the GFP gene has replaced the *GCN4* ORF in the genome. Second, the striking differences in cellular localization of Gcn4p-GFP between U and L cells are the same when *GCN4* is regulated from its native promoter and when native transcriptional/translational regulation is switched off and *GCN4* is constitutively overexpressed from the p_TEF_ promoter. Despite much higher levels of overproduced Gcn4p-GFP, the cellular Gcn4p-GFP localization pattern observed in differentiated colonies with native *GCN4* regulation was maintained: Gcn4p-GFP remained present only in the nuclei of U cells (where it is active), and disappeared from the nuclei of L cells (Fig. 2E and 3B).

### Gcn4p is inactivated in L cells by proteasomal degradation, independent of amino acid availability

The activity of Gcn4p depends not only on its expression but also on its stability (30, 33). The main mechanism described so far is the cyclin Pcl5p protein kinase Pho85p-dependent phosphorylation of Gcn4p, which leads to its ubiquitination and proteasomal degradation in the nucleus. In the absence of amino acids, Pcl5p is exchanged for Pcl7p, and Gcn4p is stabilized (30). This mechanism (which is itself independent of Gcn2p (32)) is thus controlled by amino acid availability, similar to the regulation of translation by Gcn2p. Recently, a methionine/SAM-dependent pathway was discovered that prevents Pcl5p/Pho85p-dependent Gcn4p degradation in the presence of methionine and is also independent of Gcn2p/eIF2α regulation (34). The degradation mechanism is thought to be complementary to the regulation of *GCN4* translation in yeast in response to amino acid limitation. Another biological function of Gcn4p that might be related to its stability regulation has not yet been found.

**Fig 4:**
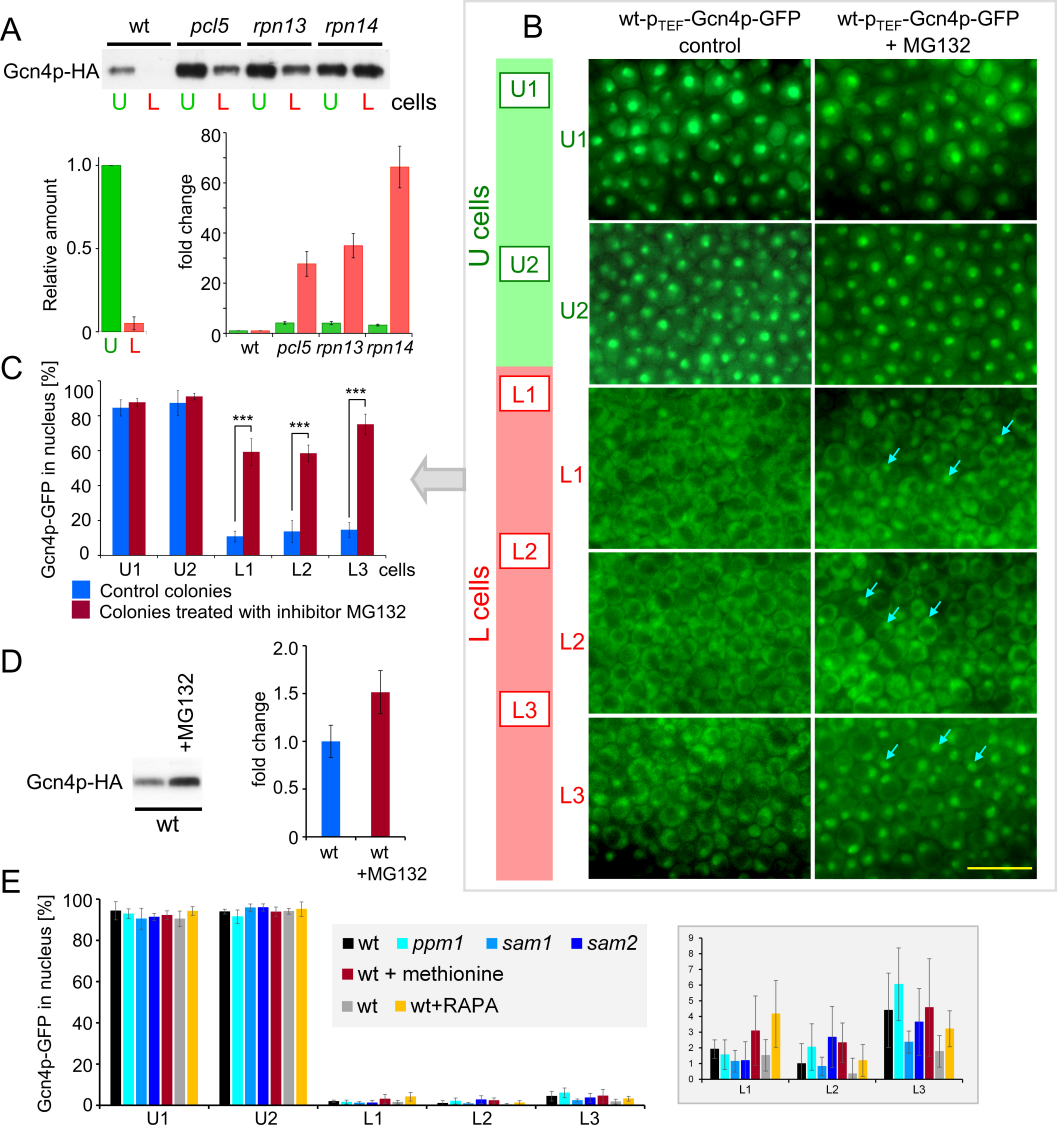
Gcn4p is inactivated in L cells in a manner dependent on proteasome activity and Pcl5p. (**A**) Differences in Gcn4p-HA levels in U and L cells of fully differentiated 15-day-old giant colonies formed by wt strain and strains in which the *PCL5*, *RPN13*, or *RPN14* genes were deleted (detected by western blots of immunoprecipitated Gcn4p-HA). A representative result from two independent biological experiments is shown. The graph shows quantification of Gcn4p-HA levels in U (green) and L (red) cells from wt and mutant giant colonies. The left graph shows the relative Gcn4p-HA amount in L cells compared with U cells of wt colonies; the right graph compares the Gcn4p amount in U and L cells of mutant colonies with the amount in the corresponding cells of wt colonies. Results are given as means and SD of four western blots from two independent experiments. Loading controls (for IP) (Fig. S5A) and Gcn4p-HA in cell lysates (without immunoprecipitation), visualized by western blot (Fig. S5B) are shown. (**B**) Effect of proteasome inhibitor MG132 on nuclear localization of Gcn4p-GFP. Nuclear localization of Gcn4p-GFP is shown on vertical cross-sections of 5-day-old microcolonies of the wt-p_TEF_-Gcn4p-GFP strain by fluorescence microscopy. The position of each field of view (right) on the colony cross-section is indicated by the same number of cell type in the rectangle on the schematic image of the colony cross-section (left). Yellow bar, 10 µm; arrows, examples of nuclear localization in L cells treated with the inhibitor. (**C**) Quantification of cells in which Gcn4p-GFP is present in the nucleus of each U and L cell subpopulation of inhibitor-treated and control colonies; fraction number U1-L3 on the x-axis corresponds to the position number on the cross-section in (**B**). Data were calculated from three independent experiments, evaluating four fields of view for each fraction and more than 600 cells per fraction in each experiment. Results are given as mean and SD. Statistical significance of differences between treated and untreated fractions was determined using an unpaired two-tailed *t*-test and GraphPad Prism 6 software; *** (*P*-value < 0.001). (**D**) Western blot of all cells from 5-day-old differentiated microcolonies of the wt-p_TEF_-Gcn4p-HA strain treated with the inhibitor MG132 and cells from wt-p_TEF_-Gcn4p-HA control colonies. Loading control in Fig. S8D. The graph shows the Gcn4p-HA WB signal in cells from colonies with MG132 expressed as a fold change compared to the signal in cells from control colonies. The mean and SD of eight western blots from five independent experiments are shown. (**E**) The cellular localization of Gcn4p-GFP in colonies is not affected by deletions of the genes *PPM1*, *SAM1,* or *SAM2*, by methionine addition, or by treatment with the TORC1 inhibitor rapamycin (RAPA). The graph shows the quantification of cells in which Gcn4p-GFP is present in the nucleus of U and L cell subpopulations (representative images are shown in Fig. **S6**). The graph on the right shows the quantification in L cells with an enlarged y-scale. The differences between wt and KO strains are not significant (determined using an unpaired two-tailed *t*-test).

Since regulation of *GCN4* expression is not important in colonies, we next focused on Gcn4p stability with respect to differential Gcn4p activity in U and L cells. The Pcl5p-Pho85p phosphorylates Gcn4p and directs it for degradation by the 26S proteasomes in the nucleus (33). Rpn13p, the subunit of the 19S regulatory particle of the 26S proteasome lid and ubiquitin receptor (47), and Rpn14p, a chaperone for assembly of the 19S regulatory particle (48, 49), play roles in this process (50). Therefore, we attempted to construct a series of strains derived from wt-Gcn4p-HA, wt-p_TEF_-Gcn4p-HA, and wt-p_TEF_-Gcn4p-GFP strains in which the *PCL5*, *RPN13*, and *RPN14* genes were deleted individually. However, whereas we successfully constructed knockout strains (KO) from wt-Gcn4p-HA with native *GCN4* expression, we failed to generate a complete collection of KO strains from wt-p_TEF_-Gcn4p-HA and wt-p_TEF_-Gcn4p-GFP strains with *GCN4* overexpression. Notably, deletion of *PCL5* failed in these strains, probably because the absence of Pcl5p further greatly increases the amount and thus the toxicity of p_TEF_-overexpressed Gcn4p-HA and Gcn4p-GFP. Therefore, in this case, we examined the effects of the deletions on the amounts of Gcn4p-HA in U and L cells from 15-day-old giant colonies of strains in which *GCN4* is controlled by the native promoter, that is, wt-Gcn4p-HA, *pcl5*-Gcn4p-HA, *rpn13*-Gcn4p-HA, and *rpn14*-Gcn4p-HA (Table 1), by western blots after concentration of Gcn4p-HA by immunoprecipitation. In contrast to wt-Gcn4p-HA colonies, in which Gcn4p-HA was very low in L cells, Gcn4p levels were greatly increased in L cells of all KO strains (Fig. 4A). In addition, Gcn4p-HA also increased in U cells of KO strains, indicating some turnover of Gcn4p in U cells as well, which is to be expected since Gcn4p is generally an unstable protein (26).

To further directly investigate whether proteasomes are responsible for the disappearance of Gcn4p from L cell nuclei, we treated developing microcolonies of the wt-p_TEF_-Gcn4p-GFP strain with the proteasome inhibitor MG132 (Z-Leu-Leu-Leu-al). To make the application of the inhibitor efficient, we grew microcolonies on filters and applied the MG132 inhibitor below the colonies at different time points in their development. Application of the inhibitor prior to differentiation into U and L cells did not affect the formation of U and L cells with typical morphology but markedly prevented the degradation of Gcn4p-GFP in the L cell nuclei (Fig. 4B and C) compared with control colonies treated in the same manner but without inhibitor. We also observed a significant increase in Gcn4p-HA levels in MG132-treated microcolonies by western blot (Fig. 4D).

Thus, two sets of results showed that proteasomal degradation of Gcn4p in L cells and stability in U cells is the main mechanism diversifying Gcn4p activity between U and L cells in differentiated colonies: (i) The amount of Gcn4p-HA (expressed from the native promoter) in L cells was significantly increased in the absence of the cyclin Pcl5p or the proteasomal subunit Rpn13p or chaperon Rpn14p. (ii) Treatment of colonies overproducing Gcn4p-GFP with a proteasome inhibitor stabilized Gcn4p-GFP in the nuclei of L cells. The fact that the difference in Gcn4p localization between U and L cells persisted even in colonies with high levels of Gcn4p-GFP (p_TEF_-overexpressed) suggests a robust proteasomal Gcn4p degradation capable of removing large amounts of Gcn4p-GFP from the nuclei of L cells.

**Fig 5:**
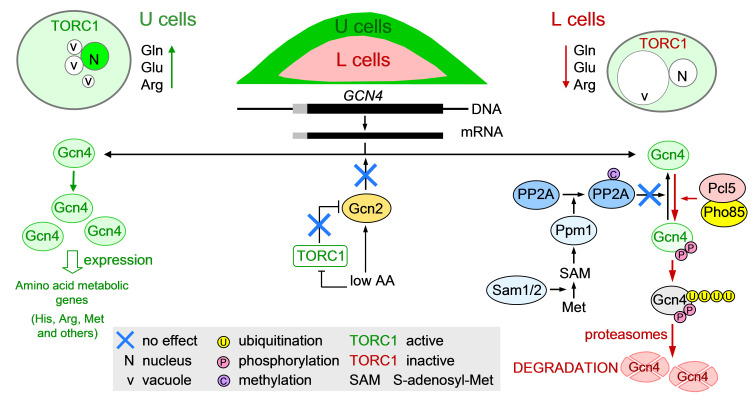
Schematic representation of the regulation of Gcn4p activity in U and L cells (description in text).

Considering other features of U and L cells, the observed diversification of Gcn4p stability in colonies is not typically dependent on amino acid availability (Fig. 5). Gcn4p is stable and active in the nuclei of U cells with active TORC1 and higher amounts of amino acids (e.g., Gln, Glu, Arg) (6, 8), whereas it is degraded and inactive in L cells with inactive TORC1 and lower amounts of these amino acids.

Moreover, our further analyses showed that the pathway of methionine-SAM-Ppm1p-PP2A- dephosphorylation and stabilization of Gcn4p in the presence of methionine (34) is not important in U and L cells (Fig. 4E and 5). The localization of Gcn4p-GFP in U and L cells was similar in wt and *ppm1*Δ *sam1*Δ, or *sam2*Δ (Table 1) colonies with disrupted components of the pathway required for methylation (and activation) of PP2A phosphatase, and the addition of methionine to wt colonies did not stabilize Gcn4p-GFP in nuclei of L cells (Fig. 4E and 5; Fig. S6), which would be expected if this pathway is involved in the stabilization of Gcn4p in colonies. On the other hand, the differential proteasomal degradation of Gcn4p between U and L cells is consistent with our findings that L cells activate transcription of genes for proteasome proteins, both 20S proteasome subunits (e.g., *PRE8*, *PUP2*, and *SCL1*) and regulatory 19S subunits (e.g., *RPN11*, *RPN13*, and *RPT6*), to a much greater extent than U cells (6). Proteasomal activity in yeast is regulated mainly at the level of expression (51), so the increased expression of proteasome subunits suggests higher proteasomal activity in L cells compared to U cells.

Gcn4p is the only AP-1 factor in yeast, but in mammals, AP-1 transcription factors are more numerous and regulated by variable conditions. *ATF4*, considered the major ortholog of *GCN4* in mammals, is an amino acid-responsive gene whose translation in mammals is activated by GCN2 upon amino acid starvation in a manner similar to *GCN4* in yeast (52, 53). However, the regulation of ATF4 is much more complex. ATF4 can also be activated in the presence of amino acids by growth-promoting signals (e.g., insulin) that also stimulate mTORC1 signaling (54–56). It is expected that mTORC1 mediates ATF4 activation independently of ISR and GCN2 (55, 57). mTORC1-ATF4-regulated genes are involved in amino acid biosynthesis, transport, and tRNA charging (56). ATF4 is also subject to proteasomal degradation regulated by environmental conditions, independent of the amount of amino acids but dependent on the amount of oxygen when ATF4 is stabilized in hypoxia (58). The functional significance is incompletely understood but is expected to be related to the positive role of ATF4 in stress response and survival and linked to regulation in cancer cells (36, 59). Compared to colonies, such regulations and conditions are reminiscent of the situation in U cells, which have sufficient amino acids and reduce mitochondrial respiration (behave like hypoxic cells) (3, 6), and in which both Gcn4p and TORC1 are active. However, in colonies treated with the TORC1 inhibitor rapamycin, we observed no changes in Gcn4p localization (Fig. 4E), indicating the independence of these regulations in U cells.

In summary, we identified a previously unknown biological function of proteasome regulation of Gcn4p activity that diversifies Gcn4p function between spatially differentiated yeast colony cells. This mechanism causes efficient degradation of Gcn4p in L cells with features of starved cells, whereas Gcn4p increases activity in U cells with various features of metabolically active cells (including amino acid content), where it contributes to the expression of genes for amino acid metabolism. Such conditions for Gcn4p activity are in contrast to the usual conditions for Gcn4p activity in liquid culture cells, which is only possible because the mechanisms of regulation of Gcn4p activity in colonies are independent of the usual regulation of Gcn4p by Gcn2p, TORC1, and amino acids. Indeed, we could not detect (Fig. S7) any differences in Gcn4p localization between two cell types in the stationary phase (quiescent and non-quiescent cells) previously identified in aging liquid cultures (4).

## MATERIALS AND METHODS

### Yeast strains and growth conditions

All yeast strains are derivatives of genetic background BY4742 and are listed in Table 1. Homologous recombination methods were used to generate knockouts in specific genes, insert p_TEF_, or construct strains with genomic GFP/HA-tagged genes. Linear DNA fragments (deletion cassettes) containing a marker gene for a specific resistance and flanked by sequences homologous to the edges of ORF (open reading frame) of the gene to be deleted or DNA fragments containing the tag sequence and marker gene homologous to the edges upstream of the stop codon of the gene to be tagged with GFP or HA were used. Transformants were selected based on the resistance to a particular antibiotic so that their other characteristics, such as auxotrophies, remained isogenic with the parent strain. Deletion cassettes for cell transformation were generated by PCR using plasmids pUG6 +25 (NatMX) and pUG6 +32 (HphMX) (10) as templates. C-terminal GFP and C-terminal HA fusion strains were constructed using the GFP-KanMX and 6HA-KanMX integration cassettes, respectively, amplified from plasmids pKT127 (60) and pYM14 (61). Strains with p_TEF_ were constructed using the Sc*TEF1* promoter integration cassette (NatMX) amplified from plasmid pYM-N20 (61). Primers used for the constructs are listed in Table S2. The position of GFP fused to individual uORFs and nAuORF2 is shown in Fig. 2A. Cells were transformed using the lithium acetate method (62).

Yeast microcolonies and giant colonies (colonies arising from a 10 μL drop of cell suspension (63)) were grown at 28°C on GMA (1% yeast extract, 3% glycerol, 1% ethanol, 2% agar, and 10 mM CaCl_2_, with or without the pH indicator bromocresol purple, BCP) at an approximate density of 5 × 10^3^ microcolonies per plate or six giant colonies per plate.

For the Q/NQ cell analyses, the wt-p_TEF_-Gcn4p-GFP strain was cultivated at 30°C with aeration in liquid YPD medium (2% yeast extract, 1% peptone, 2% glucose, 0.04 mg/mL adenine) with 50 µg/mL ampicillin according to (4).

### Microscopic analysis of the cells within the colony structure

The internal structure of the microcolonies was visualized by 2PE-CM of their vertical cross-sections embedded in agarose, as described (64). The cross-sections were placed on a coverslip, and side views of the colonies were taken with a two-photon scanning confocal microscope (SP2 AOBS MP; Leica) equipped with a mode-locked laser (Ti:Sapphire Chameleon Ultra; Coherent Inc.) for two-photon excitation and 20×/0.70 and 63×/1.20 Plan Apochromat objectives with water immersion (Fig. 1C, 2C, and E), or with confocal scanning microscope (AxioObserver.Z1) with a confocal module LSM 880 NLO and MP (Carl Zeiss, Oberkochen, Germany) with Ti:Sapphire femtosecond laser Chameleon Ultra II (Coherent, Santa Clara, CA, USA) and 25×/0.8 or 63×/1.2 water immersion LD LCI Plan or C-Apochromat objectives, respectively (images in Fig. 3B and C; Fig. S1 and S4). An excitation wavelength of 920 nm was used, and the emission bandwidth was set at 480–595 nm for GFP. Images of the microcolonies consisted of two to three composite image fields. Alternatively, the internal structure of the colonies was analyzed using cross-sections (6) (Fig. 4B; Fig. S6). After embedding the colonies in 3% agarose gel, they were sectioned using a vibrating microtome (Leica VT1200S). The sections (10–20 μm thick) were examined with a Carl Zeiss Axio Observer.Z1 fluorescence microscope with GFP filter (450–490 nm and 500–550 nm for excitation and emission, respectively) equipped with an Axiocam 506 and an Apochromat 63×/1.20W, using the software ZEN 2012 (blue edition), or Leica DMR fluorescence microscope with DIC equipped with ProgRes MF cool and a HCX PL Fluotar 100×/1.30 Oil, using the software NIS version 5.30.05.

### Microscopic analysis of the cellular localization of Gcn4p-GFP

Microcolonies of the wt-p_TEF_-Gcn4p-GFP strain were grown for 4 days at 28°C on GMA at an approximate density of 5 × 10^2^ microcolonies per plate. Cell samples were incubated with 50 µg/ml DAPI (4′,6-diamidino-2-phenylindole) solution for 5 minutes. Cells were observed using a Carl Zeiss Axio Observer.Z1 fluorescence microscope with DAPI filter (335–383 nm for excitation and 420–470 nm for emission), GFP filter (450–490 nm for excitation and 500–550 nm for emission) and DIC (to distinguish U and L cell types) equipped with an Axiocam 506 and an Apochromat 63×/1.20W, using ZEN 2012 software (blue edition).

### Cell cultivation, protein sample preparation, immunoprecipitation, and western blotting

Upper/U and lower/L cell fractions were harvested from giant colonies by micromanipulation (38). To quantify Gcn4p-HA expression in liquid cultures, cells were grown overnight at 30°C in a synthetic complete (SC) medium (0.5% ammonium sulfate, 0.14% yeast nitrogen base without amino acids and ammonium sulfate, 2% glucose) supplemented with all amino acids (SC +AA) until exponential phase. Exponentially growing cells were washed with pre-warmed, pre-aerated H_2_O, and incubated in the same medium (SC +AA), in SC +AA without histidine with 10 mM 3-aminotriazole (SC(-his)+3 AT) or in SC +AA without isoleucine/valine with 0.5 µg/mL sulfometuronmethyl (SC(-IleVal)+SM) for 1 h at 30°C.

Detection of GFP and HA-tagged proteins in the fractions was performed as described (65). Cells were disrupted with glass beads in MES lysis buffer pH 6.0 with the protease inhibitor cocktail cOmplete and PhosStop (Roche). Proteins from cell lysates or after immunoprecipitation were subjected to SDS-PAGE and transferred to a PVDF membrane. Immunoprecipitation of Gcn4p-HA from cell lysates containing 80 μg total proteins was performed (1 hour at 4°C) in PBS lysis buffer, pH 7.4 containing 8.7% glycerol, 0.1% Triton-X20, 1 mM EDTA, 2 mM DTT, 10 mM NaF, 10 mM AEBSF, cOmplete cocktail and PhosStop, using monoclonal anti-HA antibodies conjugated to agarose beads (Santa Cruz, sc-7392 AC). Beads were washed three times with the same buffer. HA (25 μg proteins/slot) and GFP (10 μg proteins/slot) in cell lysates or after immunoprecipitation were detected with rat monoclonal anti-HA (high-affinity clone 3F10, 12013819001 from Roche) or mouse anti-HA and anti-GFP antibodies (sc-7392 HRP from Santa Cruz, sc-9996 HRP from Santa Cruz) conjugated to horseradish peroxidase. The peroxidase signal was visualized using Super Signal West Pico (Pierce) on Super RX medical X-ray film (Fomei, Hradec Kralove, Czech Republic). Quantification of Gcn4p-HA was performed using UltraQuant 6.0. Membranes stained with Coomassie blue were used as loading controls (Fig. S2, S5, and S8) to normalize Gcn4p-HA or GFP signals. Statistical significance was determined using the two-tailed *t*-test and GraphPad Prism 6 software; *P* values of 0.05 or less were considered statistically significant.

### Proteasome inhibition, methionine, and rapamycin application to colonies

For proteasome inhibition, microcolonies of wt-p_TEF_-Gcn4p-GFP (for microscopy) or wt-p_TEF_-Gcn4p-HA (for Western blots) strains were grown at 28°C on a polycarbonate membrane (CycloporeTM, 47 mm diameter and pore size 0.2 µm) on a GMA plate. To inhibit proteasomal activity (according to reference (66)), the proteasome inhibitor MG132 (Z-Leu-Leu-Leu-al, Sigma, 20 µg in 20 µL 20% DMSO) was repeatedly applied to the GMA surface under the membrane (after 2, 3, and 4 days of incubation); as a control, 20 µL 20% DMSO was applied to colonies on the parallel plate. Cross-sections of 5-day-old microcolonies of the wt-p_TEF_-Gcn4p-GFP strain were examined using a Carl Zeiss fluorescence microscope with a GFP filter. The number of cells with nuclear localization of Gcn4p-GFP was determined from images of the central parts of colony cross-sections taken with a 63 × objective and expressed as a percentage of the total number of cells. Data were calculated from three independent experiments with four fields of view for each cell layer (two layers for U cells, U1 and U2, three for L cells, L1-L3; the localization of each layer is shown in Fig. 4B), and more than 600 cells per layer were evaluated in each experiment. In Fig. 4B, the contrast and brightness of the images were adjusted so that the cellular localization of Gcn4p-GFP is optimally visible in every part of the colony. The statistical significance of differences between treated and untreated fractions was determined using an unpaired two-tailed *t*-test and GraphPad Prism 6 software. *P* values of 0.05 or less were considered statistically significant. Cells from 5-day-old microcolonies of the wt-p_TEF_-Gcn4p-HA strain were used to quantify Gcn4p-HA by Western blot using a rat monoclonal anti-HA-peroxidase antibody (12013819001, Roche).

To analyze the effect of rapamycin, microcolonies of the wt-p_TEF_-Gcn4p-GFP strain were grown at 28°C on GMA. On the third day, 50 µL of rapamycin solution (250 µg/mL in 10% DMSO) was applied to the wells in the agar; 50 µL of 10% DMSO without rapamycin was used as a control. Cross-sections of 4-day-old intact colonies were examined. The number of cells with nuclear localization of Gcn4p-GFP was determined as for the proteasome inhibitor.

For methionine application, microcolonies of the wt-p_TEF_-Gcn4p-GFP strain were grown at 28°C on GMA with 200 mg/l methionine or GMA as control. Cross-sections of 5-day-old intact colonies were examined. The number of cells with nuclear localization of Gcn4p-GFP was determined as for the effect of proteasome inhibitor. Data were calculated from two independent experiments, with at least 200 cells evaluated per layer.

### Analysis of OMICs data

The intersections of genes/proteins identified as potential Gcn4p targets (Saccharomyces Genome Database, https://www.yeastgenome.org/locus/S000000735/regulation), mRNAs upregulated in U versus L cells from 15-day-old alkaline phase colonies (6, 37), and proteins upregulated in U versus L cells from 15-day-old alkaline phase colonies (38, 39) were calculated using the Venn diagram tool at https://bioinformatics.psb.ugent.be/webtools/Venn/. The genes/proteins in the intersections are shown in Fig. 1B and listed in Table S1A, respectively. Functional associations between genes/proteins whose expression is increased in U versus L cells and that belong to Gcn4p targets were analyzed using the STRING tool (https://string-db.org/). Complete STRING networks (based on data from functional and physical protein associations) of proteins within each of these two groups were constructed (Fig. 1B), using the highest confidence value (0.9) as the minimum requirement for interaction assessment and a medium (5%) false discovery rate (FDR). Functional enrichment analysis revealed gene clusters associated with Gene Ontology biological processes (GO) specific to each of the networks (Fig. 1B, Table S1B and C). FDR values represent *P* values corrected for multiple testing within each functional category using the Benjamini-Hochberg procedure.

## Data Availability

This study does not generate data deposited in external repositories. The data that support the findings of this study are available from the corresponding author upon reasonable request.

## SUPPLEMENTAL MATERIAL

Supplemental Figures and Tables mbio.00689-24-s0001.pdf Figures S1-S8; Tables S1 and S2.

ASM does not own the copyrights to Supplemental Material that may be linked to, or accessed through, an article. The authors have granted ASM a non-exclusive, world-wide license to publish the Supplemental Material files. Please contact the corresponding author directly for reuse.
