# Supplementary material for: Differential stability of Gcn4p controls its cell-specific activity in differentiated yeast colonies
Source: mBio. 2024 Apr 16;15(5):e00689-24. doi: 10.1128/mbio.00689-24 (PMC11077963; doi:10.1128/mbio.00689-24)
Supplement: Supplemental Figures and Tables — Figures S1-S8; Tables S1 and S2. [file mbio.00689-24-s0001.pdf]

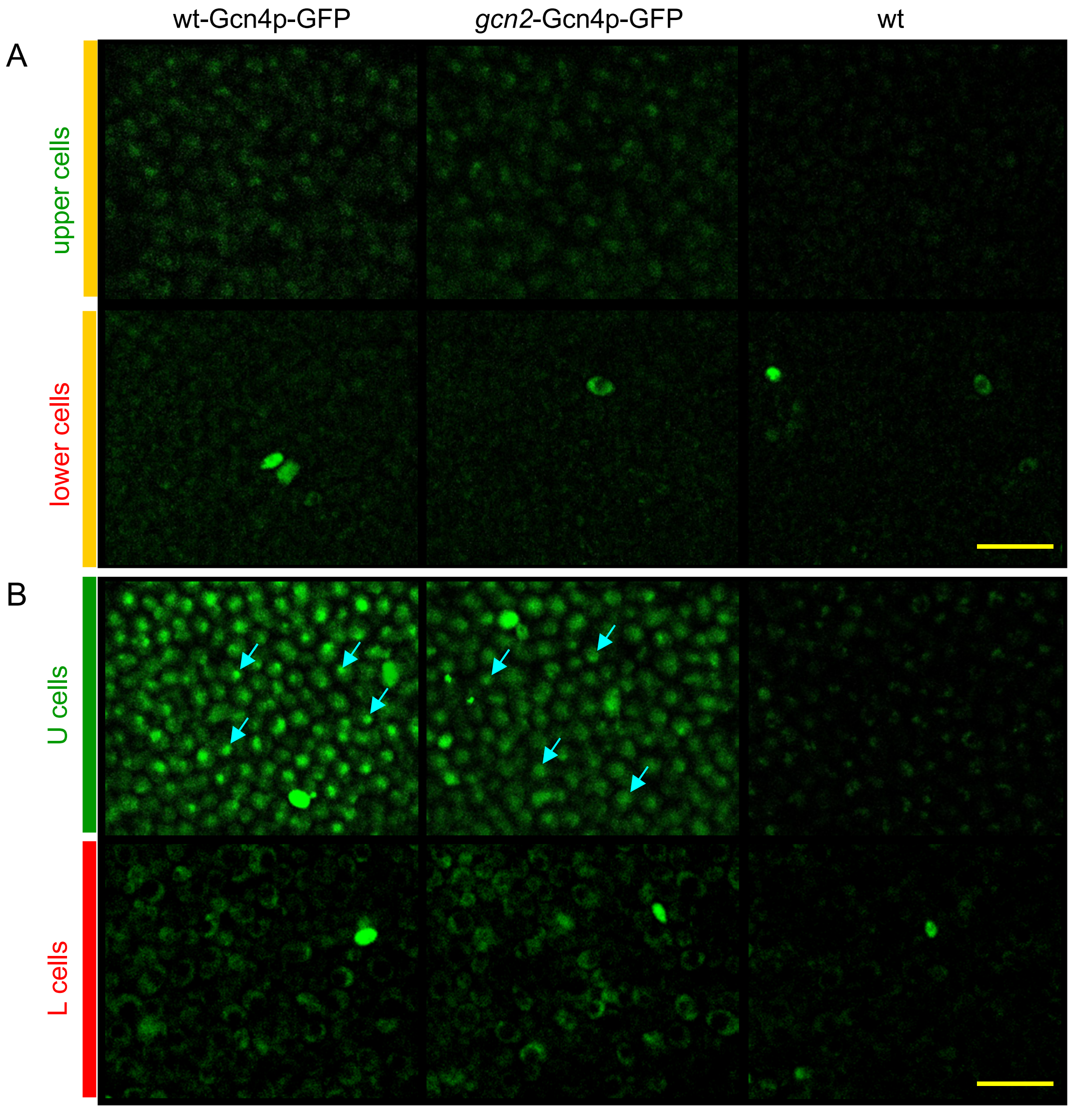

Figure S1: Gcn4p-GFP in colonies of wt-Gcn4p-GFP and *gcn2*-Gcn4p-GFP strains, compared with autofluorescence of the wt strain, using 2PE-CM of vertical cross sections (Supplementary to Figure 2E).

(A) Gcn4p-GFP in the upper and lower cells of colonies in the acidic phase (orange bars) is at the detection limit of 2PE-CM. (B) Cellular localization of Gcn4p-GFP in U (green bar) and L (red bar) cells of differentiated alkaline phase colonies. Shown is the nuclear localization of Gcn4p-GFP in U cells of the wt-Gcn4p-GFP and *gcn2*-Gcn4p-GFP strain (examples indicated by turquoise arrows). Yellow bar, 10 μm.

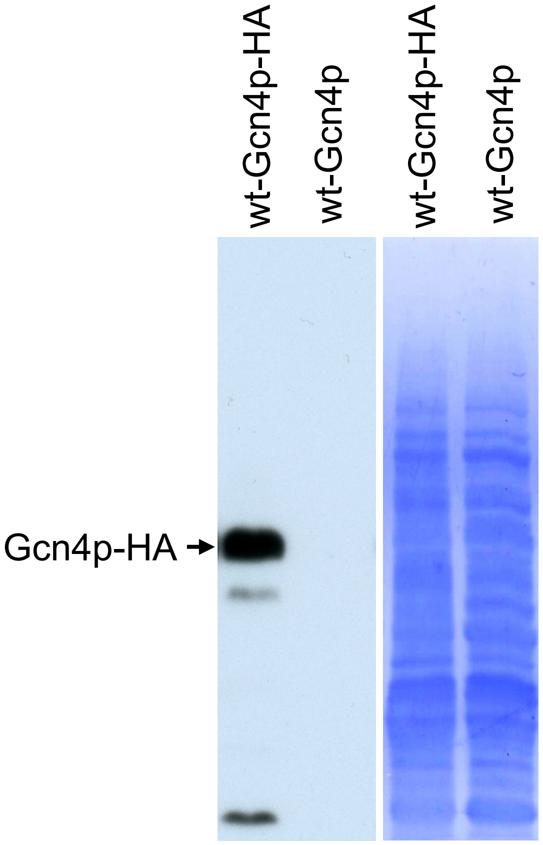

Figure S2: Anti-HA antibody specifically detects HA-tagged proteins in yeast cells. Gcn4p-HA in cell lysate compared to control - wt cells without HA tag. Left, Western blot, right, loading control (Commassie blue stained PVDF membrane).

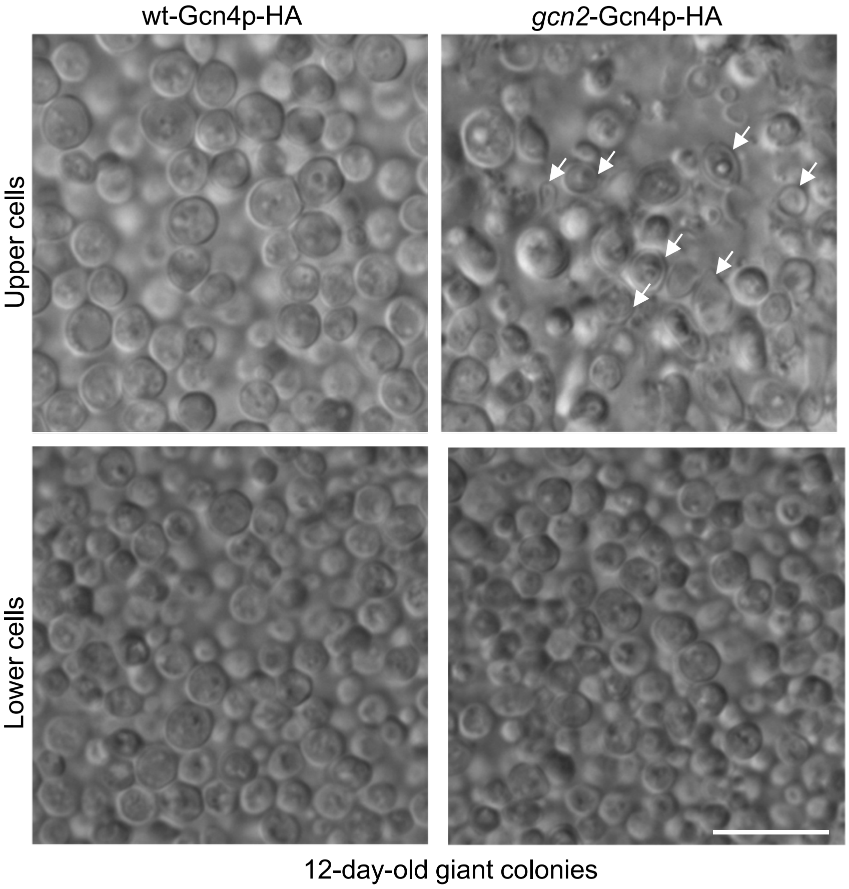

Figure S3: Dying cells in the upper parts of colonies of the *gcn2*-Gcn4p-HA strain. Cells in the upper and lower parts of wt-Gcn4p-HA and *gcn2*-Gcn4p-HA colonies, visualized by DIC on vertical colony cross-sections. Examples of dead cells in the upper part of *gcn2*-Gcn4p-HA colonies are indicated by arrows. Bar, 10 μm.

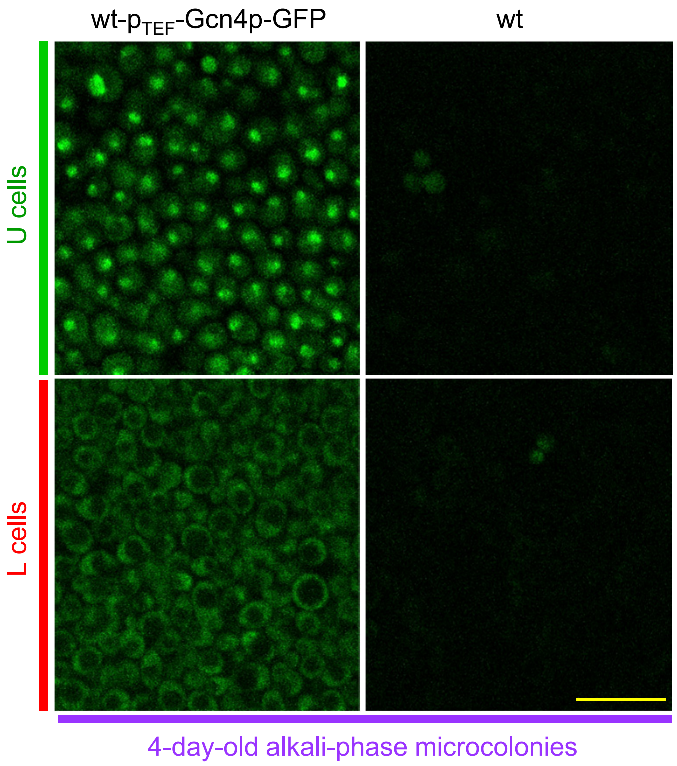

Figure S4: Comparison of Gcn4p-GFP fluorescence in U and L cells of colonies of the wt-p$_{TEF}$-Gcn4p-GFP strain with the background fluorescence of the wt strain. Bar, 10 μm**.** Supplementary to Figure 3B.

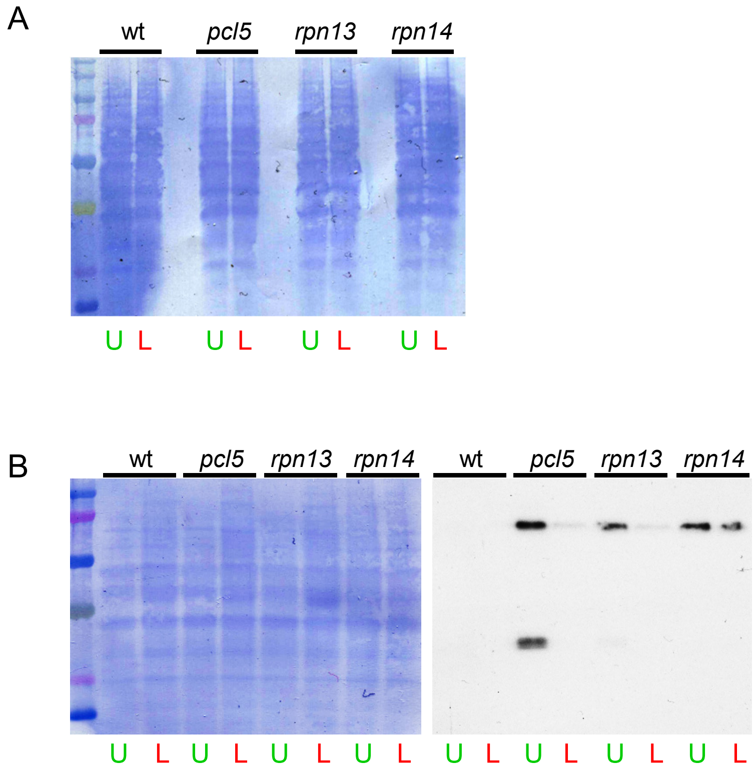

Figure S5: Loading controls for immunoprecipitation experiments and Western blot of lysates.
(A) Loading controls for IP experiment. (B) Western blot of Gcn4p-HA in lysates used for IP experiments; loading controls for WB. Supplementary to Figure 4A.

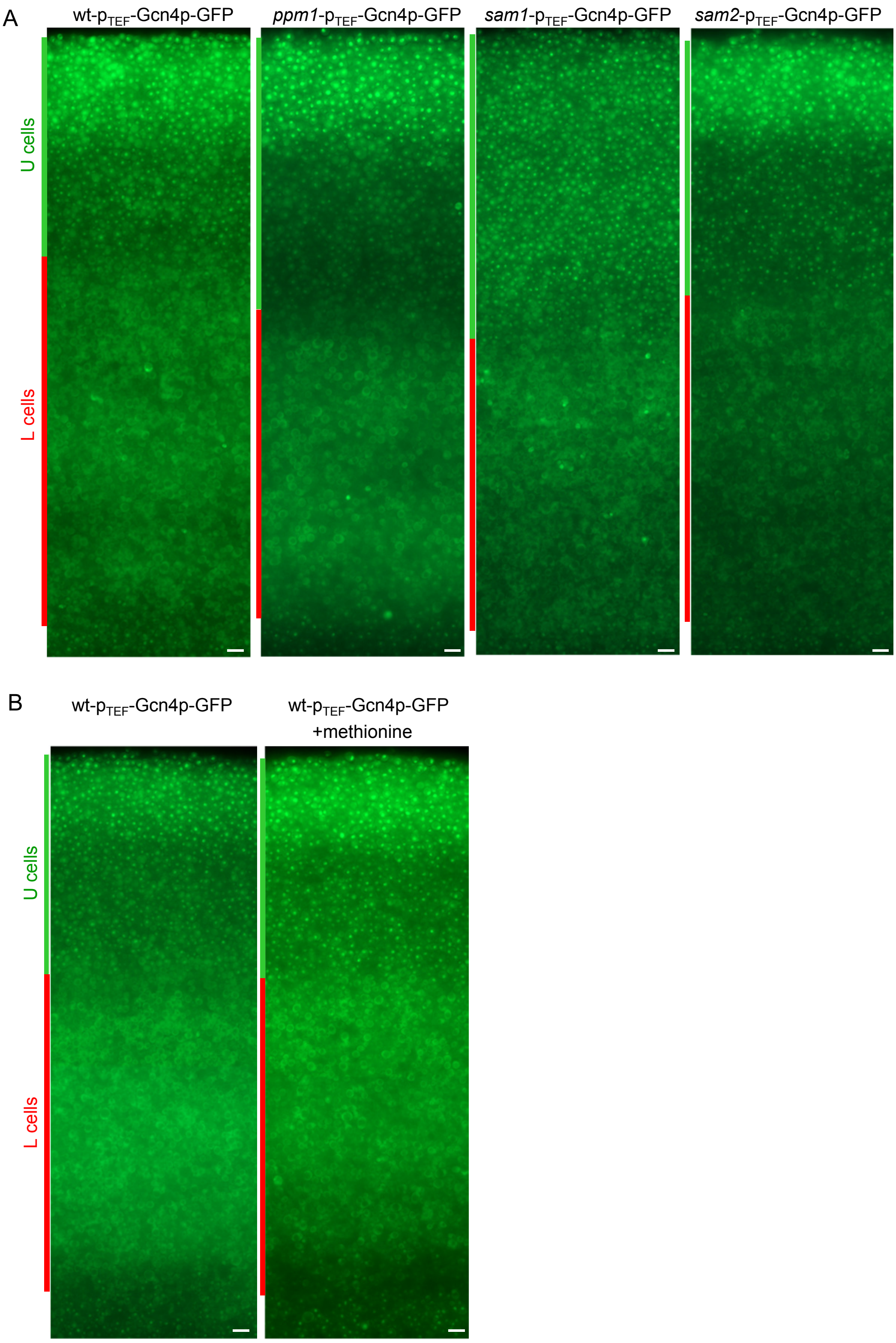

Figure S6: Cellular localization of Gcn4p-GFP in colonies of strains *ppm1*-p$_{TEF}$-Gcn4p-GFP, *sam1*-p$_{TEF}$-Gcn4p-GFP and *sam2*-p$_{TEF}$-Gcn4p-GFP and in colonies treated with methionine.

(A) Cellular localization of Gcn4p-GFP is shown on vertical cross sections of 4-day-old microcolonies of the wt-p$_{TEF}$-Gcn4p-GFP strain and derived KO strains. (B) Cellular localization of Gcn4p-GFP is shown on vertical cross sections of 4-day-old wt-p$_{TEF}$-Gcn4p-GFP microcolonies treated with methionine (right) or untreated (left). Green and red bars indicate the position of the U and L cell layers, respectively. Bar, 10 μm.

wt-p$_{TEF}$-Gcn4p-GFP

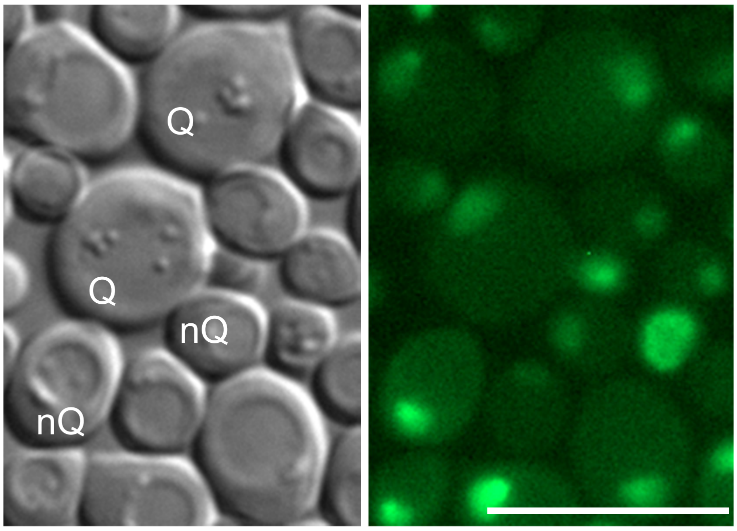

DIC Gcn4p-GFP

Figure S7: Gcn4p-GFP is present in the nuclei of both quiescent and non-quiescent cells of the liquid culture.

Cells of the wt-p$_{TEF}$-Gcn4p-GFP strain were cultured in YPD liquid medium for 4 days and then analyzed by DIC and fluorescence microscopy. Q, quiescent cells, NQ, non-quiescent cells. Bar, 10 μm.

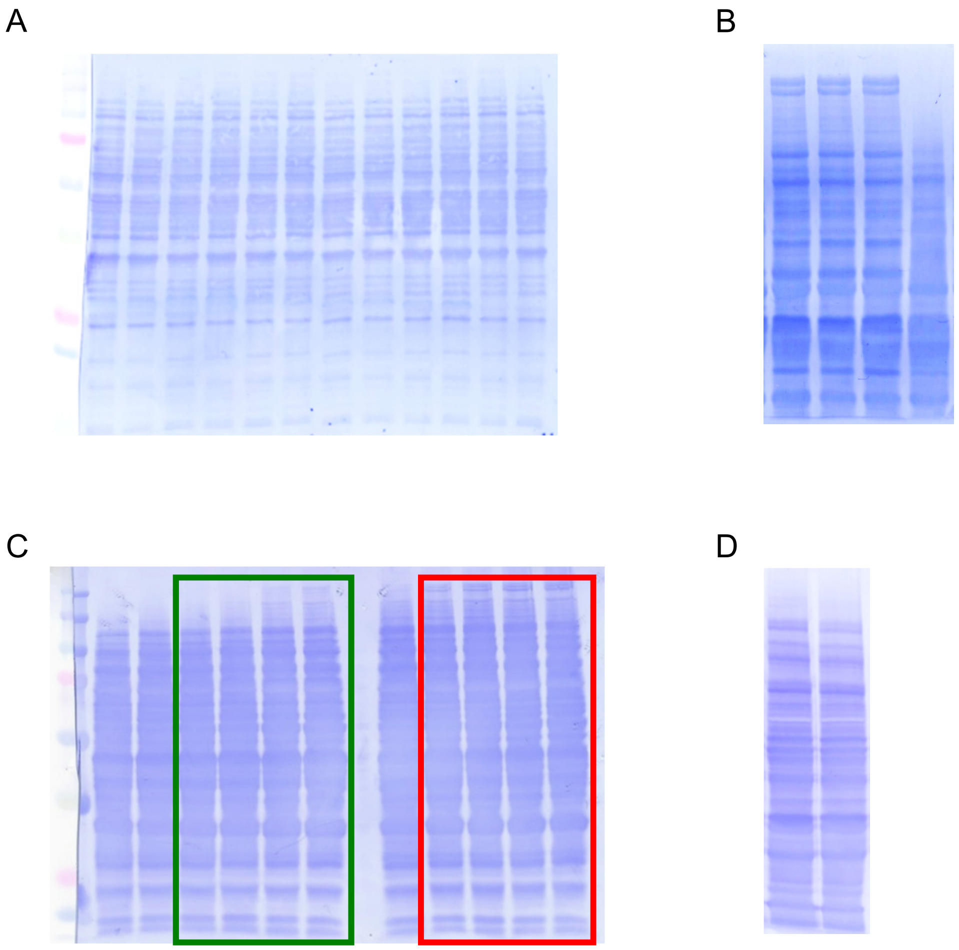

Figure S8: Loading controls
(A) for Figure 2B; (B) for Figure 2D; (C) for Figure 2F, lines for U cells in green rectangle, for L cells in red rectangle (as in Figure 2E); and (D) for Figure 4D.

Table S1: Gcn4p targets upregulated in a subpopulation of U cells

(A) Venn diagram data. Three data sets were compared: mRNAs upregulated in U cells compared to L cells (Cap et al., 2012), proteins upregulated in U cells compared to L cells (Plocek et al., 2021), and genes/proteins identified as potential Gcn4p targets (SGD, https://www.yeastgenome.org/locus/S000000735/regulation).

Overlaps	total	Genes
Gcn4p targets / U cells proteome / U cells transcriptome	19	ARG1 ARG4 ARG5,6 ARG7 ARG8 BAP2 GRE1 HIS4 HIS5 HIS7 HOM3 MET10 MET22 MSC1 SER3 SNO1 YHI9 ZPS1 ZRT1
Gcn4p targets / U cells transcriptome	41	ARO10 ARO2 ARO4 ARO8 BAT1 BIO3 BOP2 CAN1 HIS3 HSP32 ILV1 ILV5 LEU1 LEU3 LEU4 LYS1 MET13 MET16 NAT5 NIT1 NPR2 ODC2 ORT1 PEX21 PHO3 PYC2 RPL22B SAM4 SDT1 SEC53 SNZ1 SPO23 TEA1 THR4 TPN1 TRP2 TRP5 VAS1 VHT1 YIL165C YMC2
Gcn4p targets / U cells proteome	23	ADE1 ADE17 ADE57 ATO3 CAR2 CTT1 ENO1 GND2 GTT1 HIS1 LYS9 MET17 PDC6 PMA2 POT1 PPN1 SER1 SER33 SSA3 TDH1 THI6 UGA2 YGR201C
U cells proteome / U cells transcriptome	18	ACO2 APE1 ARG3 ARO3 CPA2 GCV1 HBT1 HSP104 LEU9 NDE2 NQM1 PKP2 RTS3 SRY1 STR2 STR3 TMT1 YKU70
Gcn4p targets	262	AAT2 ABF2 ACB1 ACT1 ADE12 ADE13 ADE6 ADK1 AFB1 AIM41 AIM45 AIP5 ALD5 ANS1 ARO7 ASC1 ATG34 AVT7 BIO2 CAR1 CDC39 CDC60 CLU1 CMD1 COQ11 COQ8 CTR9 CUP9 DAD3 DDR2 DLD2 DNL4 DOT5 DPB3 DPH6 DRN1 DUR3 DUT1 ECL1 ECM15 EFT2 ELO2 ELO3 EMC4 ENA1 ENO2 ERC1 ERG1 ERG11 ERG4 ERP1 ERP4 ETR1 FCY22 FKS1 FOL1 FUN26 FUR4 GCV3 GEA2 GET1 GLY1 GND1 GNP1 GRS1 GUK1 GUS1 HCS1 HSP12 HSP33 HTA2 HTB1 HTB2 HYP2 HYR1 ICL1 IOC2 IPP1 IRA2 KES1 LTP1 MAE1 MDM30 MET6 MEU1 MGA2 MIS1 MLC1 MMS1 MNL1 MOT1 MPT5 MXR2 MYO2 MYO5 NCW2 NMT1 NOC3 NRE1 NSE1 NUP159 OLA1 OST5 PAL1 PCF11 PDR12 PET122 PET18 PET9 PEX11 PEX25 PGI1 PGK1 PGM2 PHO5 PHO91 PLB3 PLP2 PMA1 PMT2 POM152 PPS1 PPX1 PRO1 PRO2 PRS4 PTA1 PTC4 PTM1 RAD3 RAP1 RIF1 ROT2 RPA135 RPL11A RPL11B RPL12B RPL15A RPL16A RPL18A RPL18B RPL19A RPL1A RPL1B RPL21A RPL21B RPL22A RPL23A RPL23B RPL24A RPL24B RPL25 RPL26A RPL26B RPL27B RPL28 RPL2A RPL2B RPL30 RPL31B RPL33B RPL34B RPL35A RPL37A RPL38 RPL42B RPL6A RPL6B RPL7A RPL7B RPL9A RPP0 RPS0A RPS0B RPS10A RPS11B RPS12 RPS14A RPS15 RPS17A RPS17B RPS18A RPS18B RPS19A RPS19B RPS1A RPS1B RPS2 RPS20 RPS22B RPS23A RPS23B RPS24A RPS24B RPS25A RPS25B RPS26A RPS27B RPS28B RPS29A RPS31 RPS4B RPS6B RPS7A RPS8A RPS8B RPS9B RTT107 SAS2 SAY1 SEC12 SEC21 SEC39 SER2 SKI2 SLX1 SMF3 SOL3 SSL1 SSM4 STB4 STE12 SUL2 SVL3 SWT21 TAL1 TAT1 TDH3 THI4 THI7 THI73 THS1 TKL1 TMH18 TSA1 TSR3 TYS1 TYW1 UBC1 UBP5 UGA4 UPA1 URK1 UTP22 VPS9 WTM1 YBR103C-A YBR220C YDR341C YGL159W YGL177W YGR067C YGR151C YHR020W YLR046C YLR297W YOR389W YPL277C YPL278C YPP1 YPQ1 YPR196W
U cells transcriptome	238	AAD10 ADE3 ADO1 ADR1 AGA2 AI5_BETA AIM17 ALD6 AMD2 ANB1 AQR1 ARG2 ARO1 ARO9 ASN1 ATG1 ATG33 ATG36 ATG41 ATP7 ATR1 BNA1 BSC1 BSC5 CDC21 CIA2 CLD1 CMC4 COA4 COX9 CPA1 CRF1 DAL7 DEF1 DIC1 DMA2 ECM13 ERR2 EUG1 FCY2 FET4 FIT3 FMP23 FOL2 FRE7 FRM2 FUS1 GAS3 GAT2 GCV2 GGC1 GID8 GIP3 GLK1 GLN3 GLT1 GPI11 HES1 HHO1 HMS2 HOM2 HOR7 HRB1 HRI1 HSC82 HSL1 HSP30 HSP42 HUB1 HVG1 HXT4 ICY1 IDP1 ILS1 ILV2 ILV3 ILV6 ISU1 ITR1 IZH2 JHD2 KAP123 KRS1 LYS12 LYS2 LYS21 MAS2 MBR1 MCT1 MDH3 MET28 MET4 MF(ALPHA)1 MGA1 MCH1 MCH4 MMP1 MND1 MNN1 MPC2 MRM1 MRP7 MRPL50 MRPS17 MRX18 MRX19 NCE103 NNF1 NOC4 NRD1 NRK1 NUT2 OAC1 OPT1 ORC3 PCL5 PDR8 PDX3 PEX2 PHO89 PIR5 PLB2 PML39 POL4 POP4 POS5 PRM5 PRM8 PSF2 PTC2 PUF3 PUG1 PUL3 PUS6 PUT3 PXP2 QDR3 RDN25-1 REE1 RFA3 RIB5 RIM9 RNP1 RPL20A RPS4A RPS9A RRI2 RRP36 RRT15 RTC2 RTG3 SAH1 SDH6 SET6 SFT2 SIP4 SKP2 SNR44 SNR64 SPC110 SPE3 SPO21 SPO24 SPO74 SPO75 SRL4 STE3 SUC2 TEC1 THI72 THR1 TPS2 TPT1 TRP3 TRP4 UBA1 UBC8 UGA3 URA2 VHR1 WAR1 YAH1 YAP1801 YBL044W YBL071C YBR027C YBR064W YBR090C YBR174C YBR190W YDL032W YDL228C YDR269C YDR444W YDR491C YER039C-A YER066C-A YER085C YER119C-A YGL024W YGL182C YGP1 YGR069W YGR161W-A YGR161W-B YHB1 YHL037C YIL059C YIL174W YJL022W YJL202C YJL218W YJR018W YJR020W YJR026W YJR027W YJR154W YKL033W-A YKL097C YKL106C-A YLL017W YLR152C YLR162W YLR235C YMC1 YMR045C YMR085W YMR1 YMR321C YNL226W YNR068C YOL160W YOR203W YOR225W YOR302W YOR366W YPL035C YPL264C
U cells proteome	174	AAC1 AAD14 ACS1 ADE2 ADE4 ADH4 ADH5 ADY2 ALB1 ALD2 ALD3 ALD4 ALT2 AMS1 APE3 ARG82 ATG23 ATG42 ATH1 ATO2 BLM10 BNA3 BUD16 BXI1 CDC19 CEM1 CIT3 CMG1 CPS1 CTA1 CVM1 CWC15 CWC27 DAK1 DAP2 DBP1 DCI1 DCS2 DGA1 DSD1 ECM10 ECM14 ECM4 EMP46 ENA1 ERR3 EXG1 FAT3 FDH1 FMP40 FMP45 FMS1 GAD1 GCY1 GDH3 GLG1 GLO1 GLO4 GPD2 GPI17 GPP1 GPX1 GRE3 GTO1 GTO3 HEF3 HSP26 HSP32 HSP78 HSP82 ICL2 ICS2 IDP3 INM2 IRC15 KKQ8 KNS1 KTI12 LAM5 LAP3 LDO16 LYS20 MAG1 MET14 MET2 MET3 MET5 MHO1 MSC3 MTD1 MXR1 NCE102 NNR1 NNR2 NPC2 NTH1 NTH2 NVJ1 OPI10 PDC5 PDR10 PHM7 PLN1 PNS1 POM33 PRB1 PRM15 PRP2 PST1 RAD52 RGI2 RNR2 RNR3 RNR4 RNY1 RRD2 RTN2 RTR1 SDH9 SEC2 SFA1 SHH3 SHH4 SHM2 SIP18 SIS1 SLT2 SOL4 SPG4 SPS100 SRX1 SSA4 SSE2 SSL2 SSO2 STE4 STI1 SYO1 TCB1 TFS1 TIP20 TKL2 TRR2 URA10 VPS33 VPS68 VTC3 VVS1 XYL2 YAP1 YDL124W YDL129W YDL218W YDR109C YDR262W YDR541C YGR130C YHR112C YIL055C YJR096W YKL050C YKL107W YLL056C YLR225C YLR326W YMR090W YMR114C YMR196W YMR315W YNL115C YNL194C YOR289W YPR127W YPR172W

Table S1: Gcn4p targets upregulated in a subpopulation of U cells

(B) Gene Ontology categories identified in mRNAs. GO categories identified in mRNAs upregulated in U cells compared to L cells (Cap et al., 2012) and belonging to Gcn4p targets (https://www.yeastgenome.org/locus/S000000735/regulation).

GO category	*term description*	*observed gene count*	*background gene count*	*false discovery rate*	*matching mRNAs in the comparison*
GO:0008652	Cellular amino acid biosynthetic process	30	131	1.47E-29	HIS7,ARO4,HIS4,THR4,HOM3,ARG5,6,SER3,ILV1,TRP2,MET10,LEU1,TRP5,MET13,ARO2,ARO8,ARG4,BAT1,HIS5,LYS1,ILV5,LEU3,ARG7,LEU4,ARG1,MET22,ARG8,ORT1,HIS3,SAM4,MET16
GO:0046394	Carboxylic acid biosynthetic process	31	186	2.31E-27	HIS7,ARO4,HIS4,THR4,HOM3,ARG5,6,SER3,ILV1,TRP2,MET10,LEU1,TRP5,MET13,ARO2,ARO8,ARG4,BAT1,HIS5,LYS1,ILV5,LEU3,ARG7,LEU4,BIO3,ARG1,MET22,ARG8,ORT1,HIS3,SAM4,MET16
GO:1901607	Alpha-amino acid biosynthetic process	28	123	2.31E-27	HIS7,HIS4,THR4,HOM3,ARG5,6,SER3,ILV1,TRP2,MET10,LEU1,TRP5,MET13,ARO8,ARG4,BAT1,HIS5,LYS1,ILV5,LEU3,ARG7,LEU4,ARG1,MET22,ARG8,ORT1,HIS3,SAM4,MET16
GO:0006520	Cellular amino acid metabolic process	33	246	3.78E-27	HIS7,ARO4,HIS4,THR4,ARO10,HOM3,ARG5,6,SER3,ILV1,TRP2,MET10,LEU1,TRP5,MET13,ARO2,ARO8,VAS1,ARG4,BAT1,HIS5,LYS1,ILV5,LEU3,ARG7,SNO1,LEU4,ARG1,MET22,ARG8,ORT1,HIS3,SAM4,MET16
GO:0044283	Small molecule biosynthetic process	35	324	2.09E-26	PYC2,HIS7,ARO4,HIS4,THR4,ARO10,HOM3,ARG5,6,SER3,ILV1,TRP2,MET10,LEU1,TRP5,MET13,ARO2,ARO8,ARG4,BAT1,HIS5,LYS1,ILV5,LEU3,ARG7,SNO1,SNZ1,LEU4,BIO3,ARG1,MET22,ARG8,ORT1,HIS3,SAM4,MET16
GO:1901605	Alpha-amino acid metabolic process	30	184	2.09E-26	HIS7,HIS4,THR4,ARO10,HOM3,ARG5,6,SER3,ILV1,TRP2,MET10,LEU1,TRP5,MET13,ARO8,ARG4,BAT1,HIS5,LYS1,ILV5,LEU3,ARG7,SNO1,LEU4,ARG1,MET22,ARG8,ORT1,HIS3,SAM4,MET16
GO:0019752	Carboxylic acid metabolic process	35	387	4.96E-24	PYC2,HIS7,ARO4,HIS4,THR4,ARO10,HOM3,ARG5,6,SER3,ILV1,TRP2,MET10,LEU1,TRP5,MET13,ARO2,ARO8,VAS1,ARG4,BAT1,HIS5,LYS1,ILV5,LEU3,ARG7,SNO1,LEU4,BIO3,ARG1,MET22,ARG8,ORT1,HIS3,SAM4,MET16
GO:0044281	Small molecule metabolic process	39	693	1.28E-20	PHO3,PYC2,HIS7,ARO4,HIS4,THR4,ARO10,HOM3,ARG5,6,SER3,ILV1,TRP2,SEC53,MET10,LEU1,TRP5,MET13,ARO2,ARO8,SDT1,VAS1,ARG4,BAT1,HIS5,LYS1,ILV5,LEU3,ARG7,SNO1,SNZ1,LEU4,BIO3,ARG1,MET22,ARG8,ORT1,HIS3,SAM4,MET16
GO:1901566	Organonitrogen compound biosynthetic process	36	869	4.31E-14	HIS7,ARO4,HIS4,THR4,HOM3,ARG5,6,SER3,ILV1,TRP2,RPL22B,SEC53,MET10,LEU1,TRP5,MET13,ARO2,ARO8,VAS1,ARG4,BAT1,HIS5,LYS1,ILV5,LEU3,ARG7,SNO1,SNZ1,LEU4,BIO3,ARG1,MET22,ARG8,ORT1,HIS3,SAM4,MET16
GO:0009066	Aspartate family amino acid metabolic process	12	72	7.14E-09	THR4,ARO10,HOM3,ILV1,MET10,MET13,ARO8,BAT1,LYS1,MET22,SAM4,MET16
GO:0009073	Aromatic amino acid family biosynthetic process	9	24	7.58E-09	HIS7,ARO4,HIS4,TRP2,TRP5,ARO2,ARO8,HIS5,HIS3
GO:0009072	Aromatic amino acid family metabolic process	10	40	1.28E-08	HIS7,ARO4,HIS4,ARO10,TRP2,TRP5,ARO2,ARO8,HIS5,HIS3
GO:1901564	Organonitrogen compound metabolic process	42	1876	2.62E-08	PHO3,HIS7,ARO4,HIS4,THR4,ARO10,HOM3,ARG5,6,SER3,ILV1,TRP2,RPL22B,SEC53,MET10,LEU1,TRP5,MET13,ARO2,ARO8,SDT1,VAS1,ARG4,BAT1,HIS5,LYS1,ILV5,LEU3,ARG7,SNO1,SNZ1,LEU4,BIO3,ARG1,MET22,ARG8,ZPS1,ORT1,HIS3,NAT5,SAM4,HSP32,MET16
GO:0009058	Biosynthetic process	41	1825	5.54E-08	PYC2,HIS7,ARO4,HIS4,THR4,ARO10,NPR2,HOM3,ARG5,6,SER3,ILV1,TRP2,RPL22B,SEC53,MET10,LEU1,TRP5,MET13,ARO2,ARO8,VAS1,ARG4,YHI9,BAT1,HIS5,LYS1,ILV5,LEU3,ARG7,SNO1,SNZ1,LEU4,BIO3,ARG1,MET22,ARG8,ORT1,HIS3,TEA1,SAM4,MET16
GO:0009067	Aspartate family amino acid biosynthetic process	10	55	1.68E-07	THR4,HOM3,MET10,MET13,ARO8,BAT1,LYS1,MET22,SAM4,MET16
GO:1901576	Organic substance biosynthetic process	40	1810	2.07E-07	PYC2,HIS7,ARO4,HIS4,THR4,ARO10,NPR2,HOM3,ARG5,6,SER3,ILV1,TRP2,RPL22B,SEC53,MET10,LEU1,TRP5,MET13,ARO2,ARO8,VAS1,ARG4,BAT1,HIS5,LYS1,ILV5,LEU3,ARG7,SNO1,SNZ1,LEU4,BIO3,ARG1,MET22,ARG8,ORT1,HIS3,TEA1,SAM4,MET16
GO:0044249	Cellular biosynthetic process	39	1764	4.51E-07	HIS7,ARO4,HIS4,THR4,ARO10,NPR2,HOM3,ARG5,6,SER3,ILV1,TRP2,RPL22B,SEC53,MET10,LEU1,TRP5,MET13,ARO2,ARO8,VAS1,ARG4,BAT1,HIS5,LYS1,ILV5,LEU3,ARG7,SNO1,SNZ1,LEU4,BIO3,ARG1,MET22,ARG8,ORT1,HIS3,TEA1,SAM4,MET16
GO:0006555	Methionine metabolic process	9	44	5.06E-07	ARO10,HOM3,MET10,MET13,ARO8,BAT1,MET22,SAM4,MET16
GO:0044272	Sulfur compound biosynthetic process	10	78	2.83E-06	ARO10,HOM3,MET10,MET13,ARO8,BAT1,BIO3,MET22,SAM4,MET16
GO:0006526	Arginine biosynthetic process	6	11	3.19E-06	ARG5,6,ARG4,ARG7,ARG1,ARG8,ORT1
GO:0009086	Methionine biosynthetic process	8	39	4.04E-06	HOM3,MET10,MET13,ARO8,BAT1,MET22,SAM4,MET16
GO:0009081	Branched-chain amino acid metabolic process	7	26	7.07E-06	ARO10,ILV1,LEU1,BAT1,ILV5,LEU3,LEU4
GO:0009082	Branched-chain amino acid biosynthetic process	6	16	1.59E-05	ILV1,LEU1,BAT1,ILV5,LEU3,LEU4
GO:0006790	Sulfur compound metabolic process	11	135	2.74E-05	PHO3,ARO10,HOM3,MET10,MET13,ARO8,BAT1,BIO3,MET22,SAM4,MET16
GO:0006807	Nitrogen compound metabolic process	47	2991	7.00E-05	PHO3,HIS7,ARO4,HIS4,THR4,ARO10,NPR2,HOM3,ARG5,6,SER3,ILV1,TRP2,RPL22B,SEC53,MET10,LEU1,TRP5,MET13,ARO2,ARO8,SDT1,VAS1,ARG4,BAT1,HIS5,NIT1,YIL165C,LYS1,ILV5,LEU3,MSC1,ARG7,SNO1,SNZ1,LEU4,BIO3,ARG1,MET22,ARG8,ZPS1,ORT1,HIS3,NAT5,TEA1,SAM4,HSP32,MET16
GO:0009064	Glutamine family amino acid metabolic process	8	62	8.09E-05	HIS7,ARG5,6,ARG4,ARG7,SNO1,ARG1,ARG8,ORT1
GO:0006551	Leucine metabolic process	5	11	0.0001	ARO10,LEU1,BAT1,LEU3,LEU4
GO:0009098	Leucine biosynthetic process	4	8	0.0013	LEU1,BAT1,LEU3,LEU4
GO:0006591	Ornithine metabolic process	4	9	0.0018	ARG5,6,ARG4,ARG7,ARG8
GO:0000105	Histidine biosynthetic process	4	10	0.0025	HIS7,HIS4,HIS5,HIS3
GO:0008152	Metabolic process	49	3661	0.0053	PHO3,PYC2,HIS7,ARO4,HIS4,THR4,ARO10,NPR2,HOM3,ARG5,6,SER3,ILV1,TRP2,RPL22B,SEC53,MET10,LEU1,TRP5,MET13,ARO2,ARO8,SDT1,VAS1,ARG4,YHI9,BAT1,HIS5,NIT1,YIL165C,LYS1,ILV5,LEU3,MSC1,ARG7,SNO1,SNZ1,LEU4,BIO3,ARG1,MET22,ARG8,ZPS1,ORT1,HIS3,NAT5,TEA1,SAM4,HSP32,MET16
GO:0006592	Ornithine biosynthetic process	3	4	0.0097	ARG5,6,ARG7,ARG8
GO:0015849	Organic acid transport	7	96	0.013	BAP2,YMC2,NPR2,CAN1,VHT1,ORT1,ODC2
GO:0071704	Organic substance metabolic process	46	3381	0.0131	PHO3,PYC2,HIS7,ARO4,HIS4,THR4,ARO10,NPR2,HOM3,ARG5,6,SER3,ILV1,TRP2,RPL22B,SEC53,MET10,LEU1,TRP5,MET13,ARO2,ARO8,SDT1,VAS1,ARG4,BAT1,HIS5,LYS1,ILV5,LEU3,MSC1,ARG7,SNO1,SNZ1,LEU4,BIO3,ARG1,MET22,ARG8,ZPS1,ORT1,HIS3,NAT5,TEA1,SAM4,HSP32,MET16

(grey shading) p value between 0.01 and 0.05

Table S1: Gcn4p targets upregulated in a subpopulation of U cells

(C) Gene Ontology categories identified in proteins. GO categories identified in proteins upregulated in U cells compared to L cells (Plocek et al., 2021) and belonging to Gcn4p targets (https://www.yeastgenome.org/locus/S000000735/regulation).

GO category	*term description*	*observed gene count*	*background gene count*	*false discovery rate*	*matching proteins in the comparison*
GO:0044281	Small molecule metabolic process	31	693	1.24E-17	ADE1,UGA2,HIS7,HIS4,PPN1,HOM3,HIS1,ARG5,6,SER3,MET10,ADE5,7,PDC6,ENO1,GND2,ARG4,SER33,HIS5,POT1,TDH1,MET17,CAR2,ARG7,SNO1,ADE17,LYS9,ARG1,MET22,ARG8,SER1,PMA2,THI6
GO:1901605	Alpha-amino acid metabolic process	21	184	1.38E-17	UGA2,HIS7,HIS4,HOM3,HIS1,ARG5,6,SER3,MET10,PDC6,ARG4,SER33,HIS5,MET17,CAR2,ARG7,SNO1,LYS9,ARG1,MET22,ARG8,SER1
GO:0043436	Oxoacid metabolic process	26	404	1.57E-17	UGA2,HIS7,HIS4,PPN1,HOM3,HIS1,ARG5,6,SER3,MET10,PDC6,ENO1,GND2,ARG4,SER33,HIS5,POT1,TDH1,MET17,CAR2,ARG7,SNO1,LYS9,ARG1,MET22,ARG8,SER1
GO:0019752	Carboxylic acid metabolic process	25	387	7.81E-17	UGA2,HIS7,HIS4,HOM3,HIS1,ARG5,6,SER3,MET10,PDC6,ENO1,GND2,ARG4,SER33,HIS5,POT1,TDH1,MET17,CAR2,ARG7,SNO1,LYS9,ARG1,MET22,ARG8,SER1
GO:1901607	Alpha-amino acid biosynthetic process	18	123	1.34E-16	HIS7,HIS4,HOM3,HIS1,ARG5,6,SER3,MET10,ARG4,SER33,HIS5,MET17,CAR2,ARG7,LYS9,ARG1,MET22,ARG8,SER1
GO:0044283	Small molecule biosynthetic process	23	324	4.68E-16	HIS7,HIS4,HOM3,HIS1,ARG5,6,SER3,MET10,PDC6,ENO1,ARG4,SER33,HIS5,TDH1,MET17,CAR2,ARG7,SNO1,LYS9,ARG1,MET22,ARG8,SER1,THI6
GO:1901566	Organonitrogen compound biosynthetic process	25	869	3.46E-09	ADE1,HIS7,HIS4,HOM3,HIS1,ARG5,6,SER3,MET10,ADE5,7,YGR201C,ARG4,SER33,HIS5,MET17,CAR2,ARG7,SNO1,ADE17,LYS9,ARG1,MET22,ARG8,SER1,PMA2,THI6
GO:1901564	Organonitrogen compound metabolic process	32	1876	2.08E-07	ADE1,SSA3,UGA2,HIS7,HIS4,HOM3,HIS1,ARG5,6,SER3,MET10,ADE5,7,PDC6,YGR201C,ENO1,ARG4,SER33,HIS5,GTT1,TDH1,MET17,CAR2,ARG7,SNO1,ADE17,LYS9,ARG1,MET22,ARG8,ZPS1,SER1,PMA2,THI6
GO:0009064	Glutamine family amino acid metabolic process	9	62	4.05E-07	UGA2,HIS7,ARG5,6,ARG4,CAR2,ARG7,SNO1,ARG1,ARG8
GO:0006525	Arginine metabolic process	6	18	5.88E-06	ARG5,6,ARG4,CAR2,ARG7,ARG1,ARG8
GO:0006591	Ornithine metabolic process	5	9	1.55E-05	ARG5,6,ARG4,CAR2,ARG7,ARG8
GO:0009058	Biosynthetic process	29	1825	2.44E-05	ADE1,HIS7,HIS4,HOM3,HIS1,ARG5,6,SER3,MET10,ADE5,7,PDC6,YGR201C,ENO1,ARG4,YHI9,SER33,HIS5,TDH1,MET17,CAR2,ARG7,SNO1,ADE17,LYS9,ARG1,MET22,ARG8,SER1,PMA2,THI6
GO:0006526	Arginine biosynthetic process	5	11	2.98E-05	ARG5,6,ARG4,ARG7,ARG1,ARG8
GO:0009070	Serine family amino acid biosynthetic process	6	27	3.66E-05	HOM3,SER3,MET10,SER33,MET17,SER1
GO:0009084	Glutamine family amino acid biosynthetic process	6	28	4.21E-05	ARG5,6,ARG4,CAR2,ARG7,ARG1,ARG8
GO:1901576	Organic substance biosynthetic process	28	1810	9.10E-05	ADE1,HIS7,HIS4,HOM3,HIS1,ARG5,6,SER3,MET10,ADE5,7,PDC6,YGR201C,ENO1,ARG4,SER33,HIS5,TDH1,MET17,CAR2,ARG7,SNO1,ADE17,LYS9,ARG1,MET22,ARG8,SER1,PMA2,THI6
GO:0008152	Metabolic process	38	3661	0.00049	ADE1,SSA3,UGA2,HIS7,HIS4,PPN1,HOM3,HIS1,ARG5,6,SER3,MET10,ADE5,7,PDC6,CTT1,YGR201C,ENO1,GND2,ARG4,YHI9,SER33,HIS5,POT1,GTT1,TDH1,MET17,CAR2,MSC1,ARG7,SNO1,ADE17,LYS9,ARG1,MET22,ARG8,ZPS1,SER1,PMA2,THI6
GO:0000105	Histidine biosynthetic process	4	10	0.00091	HIS7,HIS4,HIS1,HIS5
GO:0071704	Organic substance metabolic process	36	3381	0.0014	ADE1,SSA3,UGA2,HIS7,HIS4,PPN1,HOM3,HIS1,ARG5,6,SER3,MET10,ADE5,7,PDC6,YGR201C,ENO1,GND2,ARG4,SER33,HIS5,POT1,GTT1,TDH1,MET17,CAR2,MSC1,ARG7,SNO1,ADE17,LYS9,ARG1,MET22,ARG8,ZPS1,SER1,PMA2,THI6
GO:0044237	Cellular metabolic process	36	3421	0.0019	ADE1,SSA3,UGA2,HIS7,HIS4,PPN1,HOM3,HIS1,ARG5,6,SER3,MET10,ADE5,7,PDC6,CTT1,YGR201C,ENO1,GND2,ARG4,SER33,HIS5,POT1,GTT1,TDH1,MET17,CAR2,MSC1,ARG7,SNO1,ADE17,LYS9,ARG1,MET22,ARG8,SER1,PMA2,THI6
GO:0044249	Cellular biosynthetic process	25	1764	0.004	ADE1,HIS7,HIS4,HOM3,HIS1,ARG5,6,SER3,MET10,ADE5,7,YGR201C,ARG4,SER33,HIS5,MET17,CAR2,ARG7,SNO1,ADE17,LYS9,ARG1,MET22,ARG8,SER1,PMA2,THI6
GO:0006592	Ornithine biosynthetic process	3	4	0.0044	ARG5,6,ARG7,ARG8
GO:0009072	Aromatic amino acid family metabolic process	5	40	0.0044	HIS7,HIS4,HIS1,PDC6,HIS5
GO:0006807	Nitrogen compound metabolic process	33	2991	0.0046	ADE1,SSA3,UGA2,HIS7,HIS4,HOM3,HIS1,ARG5,6,SER3,MET10,ADE5,7,PDC6,YGR201C,ENO1,ARG4,SER33,HIS5,GTT1,TDH1,MET17,CAR2,MSC1,ARG7,SNO1,ADE17,LYS9,ARG1,MET22,ARG8,ZPS1,SER1,PMA2,THI6
GO:0044238	Primary metabolic process	34	3207	0.0062	ADE1,SSA3,UGA2,HIS7,HIS4,HOM3,HIS1,ARG5,6,SER3,MET10,ADE5,7,PDC6,YGR201C,ENO1,GND2,ARG4,SER33,HIS5,POT1,GTT1,TDH1,MET17,CAR2,MSC1,ARG7,SNO1,ADE17,LYS9,ARG1,MET22,ARG8,ZPS1,SER1,PMA2
GO:0006564	L-serine biosynthetic process	3	5	0.0064	SER3,SER33,SER1
GO:0072521	Purine-containing compound metabolic process	7	125	0.0072	ADE1,ADE5,7,ENO1,TDH1,ADE17,SER1,PMA2
GO:0055114	Oxidation-reduction process	12	457	0.0089	UGA2,HIS4,ARG5,6,SER3,MET10,CTT1,GND2,SER33,POT1,GTT1,TDH1,LYS9
GO:0009067	Aspartate family amino acid biosynthetic process	5	55	0.0144	HOM3,MET10,MET17,LYS9,MET22
GO:0006189	De novo imp biosynthetic process	3	9	0.0209	ADE1,ADE5,7,ADE17
GO:0009150	Purine ribonucleotide metabolic process	6	103	0.0219	ADE1,ADE5,7,ENO1,TDH1,ADE17,PMA2
GO:0019637	Organophosphate metabolic process	10	362	0.0286	ADE1,ADE5,7,ENO1,GND2,TDH1,SNO1,ADE17,MET22,PMA2,THI6
GO:0072522	Purine-containing compound biosynthetic process	5	75	0.0459	ADE1,ADE5,7,ADE17,SER1,PMA2
GO:0009086	Methionine biosynthetic process	4	39	0.0484	HOM3,MET10,MET17,MET22

p value between 0.01 and 0.05

Table S2: Primers

Name	Sequence	Purpose
ARO4 GFP Fw	GGCTGCTGCTGTCAGACAAAGAAGAGAAGTTAACAAGAAAGGTGACGGTGCTGGTTTA	GFP tagging of *ARO4* gene by pKT
ARO4 GFP Rv	GAGGAAAGAATGTACGTTACATATATCATTAAAAAAACATTCGATGAATTCGAGCTCG	GFP tagging of *ARO4* gene by pKT
GCN2 del Fw	TCAATAATTTTCCGTTCCCCTTAACACATACTATGTATAACAGCTGAAGCTTCGTACGC	deletion of *GCN2* gene by pUG
GCN2 del Rv	TTAACTGATGCGTTATAGCGCCGCACAGATCTTTAAAGGCGCATAGGCCACTAGTGGATCTG	deletion of *GCN2* gene by pUG
GCN4 del Fw	CAATTTGTCTGCTCAAGAAAATAAATTAAATACAAATAAACAGCTGAAGCTTCGTACGC	deletion of *GCN4* gene by pUG
GCN4 del Rv	GAGAATGAAATAAAAAATATAAAATAAAAGGTAAATGAAAGCATAGGCCACTAGTGGATCTG	deletion of *GCN4* gene by pUG
GCN4 GFP Fw	AAATGAGGTTGCCAGATTAAAGAAATTAGTTGGCGAACGCGGTGACGGTGCTGGTTTA	GFP tagging of *GCN4* gene by pKT
GCN4 GFP Rv	GAGAATGAAATAAAAAATATAAAATAAAAGGTAAATGAAATCGATGAATTCGAGCTCG	GFP tagging of *GCN4* gene by pKT
GCN4 HA Fw	TTGGAAAATGAGGTTGCCAGATTAAAGAAATTAGTTGGCGAACGCCGTACGCTGCAGGTCGAC	HA tagging of *GCN4* gene by pYM
GCN4 HA Rv	ACGAGAATGAAATAAAAAATATAAAATAAAAGGTAAATGAAATCAATCGATGAATTCGAGCTCG	HA tagging of *GCN4* gene by pYM
GCN4 nAuORF2-GFP Fw	CTACTTCGCAATCAAAACAAAATATTTTATTTTAGTTCAGTTTATGGTGACGGTGCTGGTTTA	GFP gene fusions with nAuORF2 by pKT
GCN4 nAuORF2-GFP Rv	ATTTTTCAATGATCTTTAATTTTTTAATACGATACTGATAATAACTCGATGAATTCGAGCTCG	GFP gene fusions with nAuORF2 by pKT
GCN4 uORF1-GFP Fw	TCAGTATCGTATTAAAAAATTAAAGATCATTGAAAAATGGCTTGCGGTGACGGTGCTGGTTTA	GFP gene fusions with uORF1 by pKT
GCN4 uORF1-GFP Rv	TAATTTTCTAATAATAATCTACTTTAAAAACAAAATATAATCGGTTCGATGAATTCGAGCTCG	GFP gene fusions with uORF1 by pKT
GCN4 uORF2-GFP Fw	TTTAAAGTAGATTATTATTAGAAAATTATTAAGAGAATTATGTGTGGTGACGGTGCTGGTTTA	GFP gene fusions with uORF2 by pKT
GCN4 uORF2-GFP Rv	GGACTTTAATTAATAAGGGAAAATAAATTTTCTCTTTCAATAAATTCGATGAATTCGAGCTCG	GFP gene fusions with uORF2 by pKT
GCN4 uORF3-GFP Fw	TTTGAAGAGTTATTTGTTTTGTTACCAATTGCTATCATGTACCCGGGTGACGGTGCTGGTTTA	GFP gene fusions with uORF3 by pKT
GCN4 uORF3-GFP Rv	ATTTGACAGAAAGGTAACCGTTACGGAAACATCTTGAATAAAATTTCGATGAATTCGAGCTCG	GFP gene fusions with uORF3 by pKT
GCN4 uORF4-GFP Fw	CAATTGCTATCATGTACCCGTAGAATTTTATTCAAGATGTTTCCGGGTGACGGTGCTGGTTTA	GFP gene fusions with uORF4 by pKT
GCN4 uORF4-GFP Rv	TTTTATTGGCGAGTAAACCTGGATAATTTGACAGAAAGGTAACCGTCGATGAATTCGAGCTCG	GFP gene fusions with uORF4 by pKT
GFP gcn4 Fw	TTTACCAATTTGTCTGCTCAAGAAAATAAATTAAATACAAATAAAGGTGACGGTGCTGGTTTA	p_{gcn4}GFP expression by pKT
GFP gcn4 Rv	TACACGAGAATGAAATAAAAAATATAAAATAAAAGGTAAATGAAATCGATGAATTCGAGCTCG	p_{gcn4}GFP expression by pKT
PCL5 del Fw	AATAAATATGGATGGAAATCATAGGTTTACTCCAGATTCCAAAGACAGCTGAAGCTTCGTACGC	deletion of *PCL5* gene by pUG
PCL5 del Rv	AAAGTTAATTTATTGATGAATGAAAGATATATAACATTTTCGAAAGCATAGGCCACTAGTGGATCTG	deletion of *PCL5* gene by pUG
PPM1 del Fw	GTGACTCCGCATAAACTAGATGATAAAGAGTACAAACAAGTCGCCCAGCTGAAGCTTCGTACGC	deletion of *PPM1* gene by pUG
PPM1 del Rv	CGGTAAGCATATTAAGATCAAATTAGTTGAGGCTGTAAATAAAAAGCATAGGCCACTAGTGGATCTG	deletion of *PPM1* gene by pUG
RPN13 del Fw	AACTACCTAAGTGCTGGTTGACTTATAAATTTTAAAAGAGTGTTGCAGCTGAAGCTTCGTACGC	deletion of *RPN13* gene by pUG
RPN13 del Rv	ATTTTTTCTCTTCAGTTTTTTATCAAAAAATGCCACAACCTTGATGCATAGGCCACTAGTGGATCTG	deletion of *RPN13* gene by pUG
RPN14 del Fw	AGAGCAGAAAGTAAAAAGAAATAGCGAAGTACAATAGAAACGACACAGCTGAAGCTTCGTACGC	deletion of *RPN14* gene by pUG
RPN14 del Rv	GCAGCGAAGTGAACTTTTTTGAAAAAGTTACTAGTACGAAAGAGGGCATAGGCCACTAGTGGATCTG	deletion of *RPN14* gene by pUG
SAM1 del Fw	CAGGAATTTACCCTAAAAACAAGAAAAAATAAGATAAACGAAAATCAGCTGAAGCTTCGTACGC	deletion of *SAM1* gene by pUG
SAM1 del Rv	AACATGGGAGGTTGAAGGCAGAAAAAAGTCCAAAAGGAAAAAAGCGCATAGGCCACTAGTGGATCTG	deletion of *SAM1* gene by pUG
SAM2 del Fw	TGTCTTCAATATATCCAGTATTTACGACAATATACAAACATAATCCAGCTGAAGCTTCGTACGC	deletion of *SAM2* gene by pUG
SAM2 del Rv	GAAATAATTATAAAAATCAAAATAAAACATTTATTGTCTAAATGTGCATAGGCCACTAGTGGATCTG	deletion of SAM2 gene by pUG
tefGCN4 Fw	TGTTTACCAATTTGTCTGCTCAAGAAAATAAATTAAATACAAATAAAATGCGTACGCTGCAGGTCGAC	TEF promoter exchange of *GCN4* gene by pYM
tefGCN4 Rv	GGTGAGAAACCCATTGGATTTAAAGCAAATAAACTTGGCTGATATTCGGACATGGGATCCACTAGTTCTAG	TEF promoter exchange of *GCN4* gene by pYM
